# Cystatin F Affects Natural Killer Cell Cytotoxicity

**DOI:** 10.3389/fimmu.2017.01459

**Published:** 2017-11-13

**Authors:** Milica Perišić Nanut, Jerica Sabotič, Urban Švajger, Anahid Jewett, Janko Kos

**Affiliations:** ^1^Department of Biotechnology, Jožef Stefan Institute, Ljubljana, Slovenia; ^2^Blood Transfusion Center, Ljubljana, Slovenia; ^3^The Jane and Jerry Weintraub Center for Reconstructive Biotechnology, Division of Oral Biology and Medicine, UCLA School of Dentistry, University of California-Los Angeles, Los Angeles, CA, United States; ^4^Faculty of Pharmacy, University of Ljubljana, Ljubljana, Slovenia

**Keywords:** cytotoxic cells, cystatin F, granule-mediated cytotoxicity, cathepsin C, granzyme B

## Abstract

Cystatin F is a cysteine peptidase inhibitor which, unlike other cystatin family members, is targeted to endosomal/lysosomal compartments. It is synthesized as an inactive disulfide-linked dimer which is then converted to an active monomer by proteolytic cleavage of 15 N-terminal residues. Cystatin F has been suggested to regulate the cytotoxicity of natural killer (NK) cells by inhibiting the major granzyme convertases, cathepsins C and H. To test this hypothesis, we prepared variants of cystatin F and analyzed their uptake, subcellular trafficking, and peptidase inhibition, as well as their impact on the cytotoxicity of NK-92 cells and primary NK cells. The N-glycosylation pattern is responsible for the secretion, uptake, and subcellular sorting of cystatin F in HeLa and Hek293 cells, whereas the legumain binding site had no effect on these processes. Active, N-terminally truncated, monomeric cystatin F can also be internalized by recipient cells and targeted to endo/lysosomes, affecting also cells lacking the activating peptidase. Cystatin F mutants capable of cell internalization and trafficking through the endo/lysosomal pathway significantly decreased cathepsin C and H activities, both *in situ*, following transfection and in trans, using conditioned media. Further, incubation of IL-2 stimulated NK-92 and primary NK cells with full-length and N-terminally truncated cystatin F mutants led to suppression of their granule-mediated cytotoxicity. This effect was most significant with the N-terminally truncated mutants. These results suggest that cystatin F can be an important mediator within tumor microenvironment affecting the cytotoxicity of NK cells and consequently antitumor immune response.

## Introduction

Natural killer (NK) cells are effector cells of innate immunity that play an important role in cancer immunosurveillance (1) as well as in controlling tumor growth and progression. However, functional impairments of NK cells have frequently been reported in cancer patients (2), so that understanding the complex mechanisms of the inactivation of NK cells could be of great importance for improving NK cell-based immunotherapy of cancer (3).

Although NK cells can utilize various mechanisms to eliminate target cells, granule-mediated cytotoxicity, involving the pore-forming protein, perforin, and the serine peptidases granzymes, granzymes A and B, appears to be the primary one that NK cells employ toward cancer cells (4, 5). Besides granzymes, lysosomal cysteine peptidases also play an important role in granule-mediated cytotoxicity (6, 7). Granzymes are synthesized as inactive pro-granzymes, which are proteolytically activated on reaching the secretory lysosomes (6–8). Cathepsin C is considered to be the main peptidase that is able to generate active granzyme B, whereas cathepsin H serves as an alternative one that generates active granzyme B in the absence of cathepsin C (9). Similarly, the precursor form of perforin is cleaved proteolytically by cathepsin L (10). Finally, asparaginyl endopeptidase, also known as legumain, that shares a basic mechanism of action and localization to the endo/lysosomes with lysosomal cysteine peptidases, has also been implicated in the cytotoxicity of NK cells (11).

**Figure 1:**
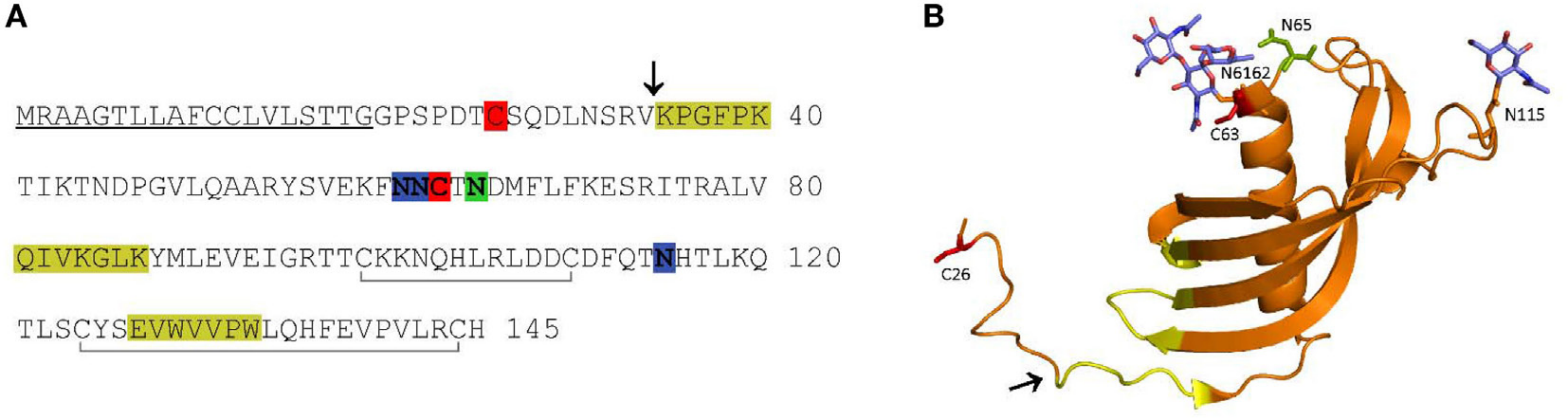
Amino acid (AA) sequence **(A)** and ribbon diagram **(B)** of human cystatin F. In the AA sequence, the signal peptide is underlined, the probable region of cysteine cathepsin interaction is highlighted in yellow, the legumain (asparaginyl endopeptidase) interaction site in green, the N-linked glycosylation sites in blue, the cysteines involved in dimerization in red, and the internal disulfide bonds indicated with gray lines below the sequence **(A)**. In the ribbon diagram (PDB 2CH9), the probable region of cysteine cathepsin interaction is indicated in yellow. The legumain interaction site (green), cysteines involved in dimerization (red) and N-linked glycans (blue) are shown as stick models **(B)**. The N-terminal truncation site is indicated with an arrow in both panels.

Major regulators of cysteine peptidases are their endogenous protein inhibitors, cystatins. The majority of these reversible and tight-binding peptidase inhibitors act extracellularly, controlling secreted or misdirected peptidases (12, 13). Cystatin F, a member of the type II cystatin family, is an exception, since it acts predominantly intracellularly. When synthesized, it dimerizes through two disulfide bridges involving Cys26 on one subunit and Cys63 on the other (Figure 1), rendering the molecule inactive as an inhibitor of cysteine cathepsins (14, 15). Cystatin F is redirected by several N-linked glycans from the secretory pathway *via* mannose-6-phosphate receptors (M6P) toward endo/lysosomal compartments (16, 17) where it is activated through monomerization. Some of the cystatin F is also secreted as an inactive dimer which can be internalized by, and activated inside recipient cells (18).

The inhibitory profile of cystatin F is dependent on its molecular form. Its disulfide-linked dimer does not inhibit the C1 family of cysteine proteases. *In vitro*, unusually strong reducing conditions are needed to dissociate the dimer (19). However, proteolytic cleavage of the 15 N-terminal amino acids (AA) significantly enhances the monomerization and also alters the inhibitory properties of the resulting monomer (20). Intact monomeric cystatin F binds tightly to cysteine endopeptidases, such as cathepsins L, F, K, and V, less tightly to cathepsins S and H, but not at all to the exopeptidases cathepsins B, X, and C (19). Cystatin F, following N-terminal truncation, becomes a strong inhibitor of cathepsin C, whereas its ability to inhibit cathepsin H is only slightly increased (20, 21). Moreover, cystatin F, in both monomeric and dimeric forms, inhibits the C13 family endopeptidase, legumain, through a distant second binding site (Figure 1) (22). Cystatin F is expressed in immune cells such as cytotoxic lymphocytes, eosinophils, neutrophils, and mast cells, as well as in dendritic cells and macrophages, most probably being involved in processes of the immune response (23–28). In dendritic cells, it is probable that cystatin F regulates the activity of cathepsin L, thus controlling the processing of procathepsin X, which promotes cell adhesion (21). Cystatin F is also implicated in several pathological processes. It has been suggested to play a role in multiple sclerosis during the acute phase of demyelination (29, 30), in polycythemia vera, a myeloproliferative disorder characterized by an increased proliferation of cells of myeloid lineages (31) and in chronic fatigue syndrome (32). Higher expression of the inhibitor correlates with the inflammatory processes associated with lung disorders (33). Increased cystatin F has also been detected in some tumors, correlating with their higher metastatic potential (34). Its overexpression and secretion was also confirmed in human cancer cell lines and patient-derived metastatic cells (34, 35). In contrast to the majority of other type II cystatins which are generally downregulated in tumors (25), cystatin F was found to be markedly upregulated in tumors of colorectal cancer patients compared to the corresponding non-tumor tissue, correlating with higher frequency of liver metastasis (36). Within the tumor microenvironment besides tumor cells several other cell types, including immune cells could be a source of increased level of cystatin F (37).

Finally, as a potent inhibitor of the pro-granzyme convertases, cathepsins C and H, cystatin F was suggested to be a regulator of the cytotoxicity of CD8+ lymphocytes and NK cells (16, 38).

In a recent study, we demonstrated that increased levels of cystatin F and decreased levels of cathepsins C and H are associated with target-induced inactivation of NK cell cytotoxicity, termed split anergy (38). This phenomenon, coupled with increased cytokine secretion and lower cytotoxicity of NK cells, can be triggered by tumor cells, monocytes or anti-CD16 antibodies. The result is decreased activation of pro-granzymes and, consequently, lower cytotoxicity, which coincides with higher concentrations of monomeric cystatin F (38–40). The aim of the present study was thus to demonstrate that cystatin F, originating from target cells, regulates *in trans* the cytotoxicity of NK cells. As an inactive dimer, secreted cystatin F is not sequestered by extracellular peptidases but is internalized by recipient cells and activated within endosomal/lysosomal vesicles. By using various mutants of cystatin F (Table 1), we analyzed the dimerization, intracellular sorting/trafficking, and peptidase inhibition, together with their impact on the cytotoxicity of NK cells. Our results point to a new mechanism, which could be used by tumor cells to escape the antitumor immune response, and suggest possible targets for improving cancer immunotherapy.

**Table 1:** Mutant forms of cystatin F, matrix DNA, and primer pairs that were used in mutagenesis.

Cystatin F variants	Plasmid name and relevant features of mutants	Matrix DNA	Primer pairs
**Wild-type**	wt cystatin F with signal sequence starting at downstream ATG	EST library clone HU3_p983D111007D2	cysF_upstream cysF_downstream
**ΔN**	pcDNA3 dNCysF Cystatin F signal sequence starting at downstream ATG and lacking the first 15 N-terminal residues (N-terminally truncated)	pcDNA3 cystatin F with signal sequence starting at downstream ATG	dNCysF-F dNCysF-R
**ΔN C63S**	pcDNA3 dN C63S CysF N-terminally truncated with C63S mutation	pcDNA3 dNCysF	dNCysF-F dNCysF-R
**N6162S**	pcDNA3 N6162SCysF Double N-linked glycosylation sequence mutant −61 and 62 Asn residues altered to Ser	pcDNA3 cystatin F with signal sequence starting at downstream ATG	cysF_N61-62S_F cysF_N61-62S_R
**N6162S N115Q**	pcDNA3 N6162S N115QCysF Triple N-linked glycosylation sequence mutant	pcDNA3 N6162SCysF	CysF-N96Q CysF-N96Qc
**N65K**	pcDNA3 N65K CysF legumain binding sequence mutant	pcDNA3 wt cystatin F with signal sequence starting at downstream ATG	CysF-N65K F CysF-N96Q R
**N65K N6162S N115Q**	pcDNA3 N65K N6162S N115Q CysF Triple N-linked glycosylation and legumain binding sequence mutant	pcDNA3 N6162S N115QCysF	CysF-N65K F CysF-N96Q R
**ΔN N65K**	pcDNA3 dN N65Q CysF N-terminally truncated and legumain binding sequence mutant	pcDNA3 dNCysF	CysF-N65K F CysF-N96Q R
**wt-His**	pcDNA3 WT CysF 5His His-tagged wt cysF	pcDNA3 wt Cystatin F	cysF-6H-pCDNA_F cysF-6H-pCDNA_R
**N6162S N115Q-N6162S N115Q His**	pcDNA3.1 N6162S N115Q CysF 5His His-tagged N6162S N115Q	pcDNA3 N6162S N115Q CysF	cysF-6H-pCDNA_F cysF-6H-pCDNA_R
**N65K-His**	pcDNA3 N65K CysF 5His His-tagged N65K	pcDNA3 N65K CysF	cysF-6H-pCDNA_F cysF-6H-pCDNA_R
**ΔN-His**	pcDNA3 dNCysF 5His His-tagged ΔN	pcDNA3 dNCysF	cysF-6H-pCDNA_F cysF-6H-pCDNA_R
**ΔN N65K-His**	pcDNA3 dN N65Q CysF 5His His-tagged ΔN N65K	pcDNA3 dN N65Q CysF	cysF-6H-pCDNA_F cysF-6H-pCDNA_R

## Materials and Methods

### Cell Culture

Hek293 (ATCC: CRL-1573), HeLa (ATCC: CCL-2), and MCF-7 (ATCC: HTB-22) cells were maintained in DMEM media supplemented with 10% fetal bovine serum (10270-106, Gibco). K562 (ATCC: CCL-243) cells were grown in RPMI 1640 culture medium (12-115F/12, Lonza) supplemented with 10% heat inactivated fetal bovine serum (hiFBS) (10500-064, Gibco). The human IL-2-dependent NK-92 cells (ATCC: CRL-2407) were maintained in RPMI 1640 culture medium, supplemented with 12.5% hiFBS, 12.5% Heat Inactivated Horse Serum (Gibco, 26050088), and 100 U/mL rhIL-2 (H-7365.0050, Bachem). NK-92 can be genetically altered to target specific tumor antigens and express activating receptors, thereby increasing their cytotoxicity and potential for use in immunotherapy (41). All cell culture media contained 100 U/mL penicillin and 0.1 mg/mL streptomycin (15140122, Gibco). Suspension freestyle Hek293F cells (K900001, Thermo Fisher Scientific) were grown in FreeStyle Hek293 Expression Medium (12338018, Thermo Fisher Scientific). Apart from FreeStyle Hek293, which were grown in a humidified 37°C incubator on constant rotation and under 8% CO_2_, the rest of cell lines and primary NK cells were grown in a humidified 37°C incubator under 5% CO_2_.

### Construction, Expression, and Purification of Mutant Forms of Cystatin F

The cystatin F (*CST7*) coding sequence (starting at downstream genomic ATG codon) was amplified from a plasmid from EST library clone HU3_p983D111007D2 (ImaGenes, Berlin, Germany) using primers for ligation into vector pcDNA3 (Table 1; Table S1 in Supplementary Material). This sequence served as the basis for the design of mutagenic oligonucleotides (Table 1; Table S1 in Supplementary Material). The amplified fragment and the target plasmid were digested with *Hind* III (R3104M)/*Eco*RI (R3101M) for cloning into pcDNA3, and ligated using T4 DNA ligase (M0202L) all purchased from NewEnglandBiolabs. N-terminally truncated cystatin F, double unglycosylated mutant (N6162S) and cystatin F with mutated binding site for legumain (N65K) were generated in PCR site-directed mutagenesis using KOD Hot Start DNA Polymerase (71086-5, Novagen Inc), expression plasmid pcDNA3 with cystatin F sequence as template and different pairs of primers (Table 1; Table S1 in Supplementary Material). The *Dpn*I endonuclease (ER1705, Fermentas) was used for digestion of the matrix sequence and recovery of the vectors containing mutated inserts (42). Double (N6162S) unglycosylated and non-glycosylated (N6162SN115Q) mutants were prepared similarly using single or double mutants as matrix DNA in mutagenic PCR. For protein expression in Hek293 FreeStyle cells, additional mutagenesis was performed to introduce a 5-histidine C-terminal tag (Table 1; Table S1 in Supplementary Material) and single, double, or triple mutants as matrix DNA.

The endotoxin free pcDNA3 plasmids, containing C-terminally 6-His tagged full-length (wild-type) and mutant species of cystatin F, were prepared using NucleoBond Xtra Midi EF kit (740422.50, Macherey-Nagel). Transient transfection of freestyle Hek293F cells was performed using 293fectin™ Transfection reagent (K900001, Thermo Fisher Scientific) and following the manufacturer’s instructions. Transfected cells were grown for 1 week; the cells were then pelleted, the cell culture medium concentrated using Amicon^®^ Ultra-15 Centrifugal Filter Unit with Ultracel^®^-3 membrane (UFC900324, Merck Millipore) and used for protein purification. The NaCl concentration in samples was adjusted to 400 mM and the medium was passed over TALON metal affinity resin (635502, Clontech Laboratories, Inc.) equilibrated with 50 mM Tris, 400 mM NaCl, pH 7.4. The resin was washed with 50 mM Tris, 500 mM NaCl, 10 mM imidazole, pH 7.4 and the proteins eluted with 50 mM Tris, 120 mM NaCl, 400 mM imidazole, pH 7.4. Eluates were concentrated using Amicon^®^ Ultra-15 Centrifugal Filter Unit (UFC900324, Millipore), and the buffer exchanged to 20 mM Bis-Tris pH 6.5. The recombinant proteins were further purified using ÄKTA FPLC system (GE Healthcare Lifesciences) controlled by Unicorn software version 5.11 (GE Healthcare, Piscataway, NY, USA). The Mono S HR 5/5 column (17-0547-01, Pharmacia) was equilibrated with five column volumes (CV) of 20 mM Bis-Tris pH 6.5 buffer prior to loading, washed after loading with 3CV of the same buffer, followed by 15 CV of 0–1 M NaCl linear gradient elution at pH 6.5 in 20 mM Bis-Tris. The resulting protein was immediately dialyzed against phosphate buffered saline (PBS) and concentrated using Amicon^®^ Ultra-15 Centrifugal Filter Unit. The purity of isolated recombinant proteins was verified by SDS–PAGE and Silver staining (Figure S3 in Supplementary Material).

### Transfection and Protein Uptake

Western blot analysis of cystatin F and the inhibition of intracellular peptidases was performed on post-nuclear lysates of HeLa and Hek293 cells upon transfection with pcDNA3 containing different untagged cystatin F mutants and upon conditioning (exposure to cell culture medium from cells transfected with vector containing cystatin F mutants for 24 h). Cells (HeLa and Hek293) were grown in tissue culture treated 6-well plates (protease assays) or 24-well plates (immunoblot analysis) and transfected with plasmids containing either wild type or mutant forms of cystatin F, using PolyJet DNA Transfection Reagent (SL100688, Sinangen), following manufacturer’s instructions. Transfected cells were left in culture for 24 h after which the cystatin F-enriched medium was removed, centrifuged (5 min, 500 *g*) and added to non-transfected cells (conditioning-internalization assays) which were grown for an additional 24 h before analysis. Cells transfected with empty pcDNA3 vector and conditioned with medium from empty vector-control cells served as controls.

### Immunoflourescence Staining

For immunofluorescence staining, HeLa and Hek293 cells were grown on Poly-l-lysine-coated coverslips, transfected using PolyJet DNA Transfection Reagent (SL100688, Sinangen), following the manufacturer’s instructions, then grown for another 24 h. Cell media were removed, centrifuged, and used in internalization assays for the non-transfected cells. The cells were washed with PBS, fixed with 4% paraformaldehyde (15710, Electron Microscopy Sciences) in PBS (10 min), followed by 10 min permeabilization in 0.1% Triton X-100 in PBS. Non-specific staining was blocked with 3% BSA in PBS for 1 h. Cells were co-stained with primary, affinity-purified rabbit anti-cystatin F, mouse monoclonal anti LAMP-1 antibody (ab25630, Abcam) and goat polyclonal anti Golgin-97 (sc-74632, Santa Cruz Biotechnology) and secondary antibodies, donkey anti-mouse conjugated with Alexa Fluor 488 (2.5 µg/mL, A-21202, Thermo Fisher Scientific), donkey anti-rabbit antibodies conjugated with Alexa Fluor 546 (2.5 µg/mL, A-10040, Thermo Fisher Scientific), donkey anti-goat antibodies conjugated with Alexa Fluor 633 (2.5 µg/mL, A-31570, Thermo Fisher Scientific), each time for 1 h in the presence of 3% BSA in PBS. Cells were washed three times with PBS after each of these steps. Lysotracker Red DND (L7528, Thermo Fisher Scientific) was purchased from Life Technologies. For cystatin F staining, affinity-purified rabbit anti-cystatin F antibody (Davids Biotechnologie, GmbH, Germany) raised against recombinant human cystatin F was used unless stated otherwise.

For internalization assays, HeLa and Hek293 cells were grown on glass coverslips for 12 h before the addition of cystatin F-containing conditioned media from transfected cells or of control media, grown for an additional 24 h and stained as described above. Cells were mounted on glass slides with Prolong Gold Antifade Reagent containing nuclear 4′,6-diamidino-2-phenylindole stain (P36935, Thermo Scientific). Cells were imaged using an LSM-710 confocal microscope (Carl Zeiss, Germany) using a 63 PlanFluar/NA 1.45 objective and ZEN 2010 B SP1 software (Carl Zeiss) followed by processing with ZEN 2010 B SP1 software. Images were obtained by sequential excitation with Diode (405-30), Argon (488), HeNe (543), and/or HeNe (633) lasers, using a separate channel for a specific fluorophore in order to avoid cross-talk between different fluorophores.

### Immunoblotting and Protease Assays

Glycosylation status of cell culture medium of HeLa cells transfected with pcDNA3 containing different untagged cystatin F mutants was analyzed with N-glycosidase F (PGNase; Roche). The samples were prepared according to manufacturer’s instructions. Briefly, 4 µg of cell culture media were mixed with reaction buffer (Roche) and boiled for 10 min at 100°C. Once cooled, 3 U of N-glycosidase F (PGNase) was added and the reaction was incubated overnight at 37°C. For western blot analysis, the cells were detached using mild dissociating reagent TrypLE Select (12563029, Gibco), washed once with ice-cold PBS, and lysed (10^8^ cells/mL RIPA buffer: 0.15 M NaCl, 1% TritonX-100, 0.1% SDS, 50 mM Tris, pH 8.0), supplemented with protease and phosphatase inhibitor cocktail tablets (PhosSTOP with cOmplete and cOmplete ULTRA Protease Inhibitor Tablets, Roche, Switzerland). NK-92 and primary NK cells were washed twice in ice-cold PBS and lysed under the same conditions as described above. Protein concentration was determined with DC Protein Assay (5000116, Bio-Rad Laboratories). Immunoblotting proteins were separated by 12 and 15% SDS-PAGE as described (43). Membranes were blocked for 1 h in 5% non-fat dry milk in TBS with 0.5% Tween-20 (for anti-Granzyme B antibody) or with PBS for other antibodies. Primary antibodies diluted in blocking solution were incubated overnight at 4°C. Antibody-reactive proteins were detected by incubation, in blocking solution for 1 h, with horseradish peroxidase (HRP)-conjugated secondary antibodies (anti-rabbit-HRP, 111-035-045, Jackson ImmunoResearch or anti-mouse-HRP, 715-035-150, Jackson ImmunoResearch) or in the dark with fluorescent secondary antibodies [goat anti-mouse IgG (H + L)-DyLight 550, 84540 or goat anti-rabbit IgG (H + L)-DyLight 650, 84546, Invitrogen]. Membranes with HRP-conjugated antibodies were visualized with LumiLight Plus Western Blotting substrate (12015196001, Roche). Images were acquired using a GelDoc System (Bio-Rad), processed, and quantified using Image Lab Software (Bio-Rad). We used antibody raised against 63 AA in the C-terminal part of cystatin F (HPA040442, Sigma-Aldrich) for analysis of NK and NK-92 cell lysates, culture media and cell lysates (Hek293 and HeLa cells) upon transfection and internalization. Other antibodies used in western blots include: mouse anti-β-actin (ab8226, Abcam), mouse monoclonal antibody for Granzyme B (sc-8022, Santa Cruz Biotechnology), and mouse anti-GAPDH antibody (sc-32233, Santa Cruz Biotechnology).

For protease assays the cells were washed in PBS and detached using TrypLE Select (Invitrogen). All subsequent steps were carried out at 4°C. Cells were centrifuged (300 *g*) and washed in PBS. Cell pellets were then resuspended in lysis buffer containing either 50 mM citrate buffer, pH 6.2 with 1% Triton-X-100 (for cathepsins) or in 25 mM HEPES, pH 7.4 with 250 mM NaCl, 2.5 mM EDTA, 0.1% NP-40 (for granzymes) and incubated for 15 min. Lysed cells were then centrifuged (14,000 *g*) for 20 min to obtain post-nuclear cell lysates; the pellets were discarded. Protease activity was determined by following the hydrolysis of their respective substrates. The rate of hydrolysis was followed in assay buffer containing 10 µg of post-nuclear cell lysates and specific substrates. Each lysate was analyzed in triplicate. The following activation buffers were used: 100 mM MES, 2 mM EDTA, 5 mM dithiothreitol (DTT), pH 6.5 (for cathepsins L and H), 25 mM MES, 100 mM NaCl, 5 mM DTT, pH 6 (cathepsin C) and 39 mM citric acid, 121 mM Na_2_HP0_4_, 1 mM EDTA, 5 mM DTT pH 5.8 (for legumain), 50 mM Tris, 100 mM NaCl pH 7.4 (Granzyme B) and 20 mM Tris, 150 mM NaCl, pH 8.1 (Granzyme A). Post-nuclear cell lysates were incubated in black 96-well plates for 15 min in assay buffer before the addition of substrates:H-Gly–Phe-7-Amido-4-methylcoumarin (AMC) for cathepsin C (70 µM, I-1220.0050, Bachem), H-Arg-AMC for cathepsin H (20 µM, I-1050.0250, Bachem), Z-Phe–Arg-AMC for cathepsin L (50 µM, I-1160.0050 Bachem), benzoyloxycarbonyl-Ala–Ala–Asn-AMC (12 µM, I-1865.0050, Bachem) for legumain, acetyl-Ile–Glu–Pro–Asp-AMC for granzyme B (50 µM, I-1835.0005, Bachem) and benzoyloxycarbonyl Gly–Pro–Arg-AMC for granzyme A (200 µM, I-1150.0025, Bachem). Fluorescence was quantified by measuring (for 30 min) excitation at 370 nm and emission at 460 nm on microplate reader Infinite M1000 (Tecan). Protease activities are expressed as the rate of AMC release over time.

### Isolation of Peripheral Blood NK Cells

Peripheral blood was obtained from healthy volunteers at the Blood Transfusion Center of Slovenia, according to institutional guidelines. Samples were collected following local ethics committee approval (0120-279/2017-3). Peripheral blood lymphocytes were obtained after Ficoll-hypaque centrifugation and enriched in NK cells by negative selection using the NK Isolation Kit from Miltenyi Biotec (130-092-657) according to the manufacturer’s instructions. The purity of NK cell population was found to be greater than 98%, based on flow cytometric analysis of anti-CD3 (300319, BioLegend) and anti-CD56 antibody (130-090-755, Miltenyi Biotech) labeling. NK cells were either used directly, or cultured with or without IL-2 (1,000 IU/mL) for 48 h at 37°C and 5% CO_2_. The levels of contaminating CD3+ T cells remained low, at 0.7 ± 0.01%, similar to those obtained by non-specific staining using isotype control antibody (400147, BioLegend) throughout the experimental procedures. Flow cytometry was performed using the FACSCalibur flow cytometer (Becton Dickinson Immunocytometry System, San Jose, CA, USA) for acquisition and FlowJo software (TreeStar) for sample analysis.

### Cytotoxicity Assay

Cytotoxicity was measured using the Total Cytotoxicity Detection Kit (972, Immunochemistry) based on a flow cytometric analysis, using K562 cells or MCF-7 cells as targets, and NK-92 or primary NK cells as effectors. Target (K562 or MCF-7) cells were fluorescence labeled with carboxyfluorescein succinimidyl ester (CFSE) and added to 96-well flat-bottomed polypropylene microtiter plates (Costar, Corning Life Science). The effector cells were then added to achieve different target: effector cell ratios (1:1, 1:2, 1:5, 1:10, 1:20 for NK-92 cells or 1:0.1, 1:0.5, 1:1, 1:2, 1:5 for primary NK cells). Various cystatin F mutants (80 nM final concentration) were added to the cell mixture. The samples were incubated for 4 h at 37°C in 5% CO_2_, then sulforhodamine-fluorochrome labeled inhibitor of caspases (SR-FLICA) was added to all samples and the cells incubated for another 45 min at 37°C. After incubation, the cocultured cell populations of effector and target cells were transferred to round-bottom polystyrene tubes (Falcon, ThermoScientific). Viability stain 7-aminoactinomycin D (7-AAD) was added following the manufacturer’s instructions and the samples were acquired within 30 min of staining. All samples were acquired using a FACSCalibur flow cytometer (Becton Dickinson Immunocytometry System, San Jose, CA, USA). Sample acquisition and flow cytometer compensation was done according to the manufacturer’s instructions, acquiring 9,000 of target cells per sample. Sample analysis was performed using FlowJo softwear (TreeStar) as previously described (44). Briefly, target cells were gated by their side scatter and FL-1 fluorescence (CFSE-positive staining, FL-1) and 7-AAD uptake (FL-3) and SR-FLICA (FL-2) staining was determined within the gated cells. To determine spontaneous lysis of target cells, samples without effector cells (effector: target concentrations of 0:1 as negative control) were included in the experiments and spontaneous lysis of target cells was subtracted from the cytotoxic percentage obtained for each sample to give the net cytotoxicity percentage. LU 30/10^6^ cells were calculated using the inverse of the number of effector cells needed to lyse 30% of target cells × 100 (38). When testing the effects of anti-cystatin F antibody on cytotoxicity of NK-92 cells, the cells were activated and mixed with stained target cells as described above, incubated with the antibody (in final concentration 200 mM) with or without the addition of wild-type cystatin F. After the incubation the cells were further stained and analyzed as described above.

For flow cytometric analysis of exocytosis in the presence of recombinant cystatin F NK-92 cells were activated as described above, incubated with cystatin F mutants (80 nM final concentration) for 4 h and receptor activation independent lytic granule exocytosis was induced by incubation with DAG analog, phorbol myristate acetate (Sigma) (50 nM final concentration), and ionomycin (Sigma) (1 µM final concentration) for 1 hr at 37ºC. Exocytosis was assayed by measuring the binding of anti-CD107a (LAMP-1)-VioBright FITC, human (130-106-233, Miltenyi Biotech) antibody to the externalized lysosome-associated membrane protein 1 (LAMP-1).

### Statistical Analysis

Data were analyzed using GraphPad Prism 5 software. The values represent mean ± SD of the values obtained. For kinetic data the slopes of linear regression curves were calculated and statistical differences in all experiments were determined using a one-way ANOVA. Statistical significance was accepted when *P* < 0.05.

## Results

### Subcellular Localization, Secretion, and Uptake of Full-Length Cystatin F and Its Mutants

**Figure 2:**
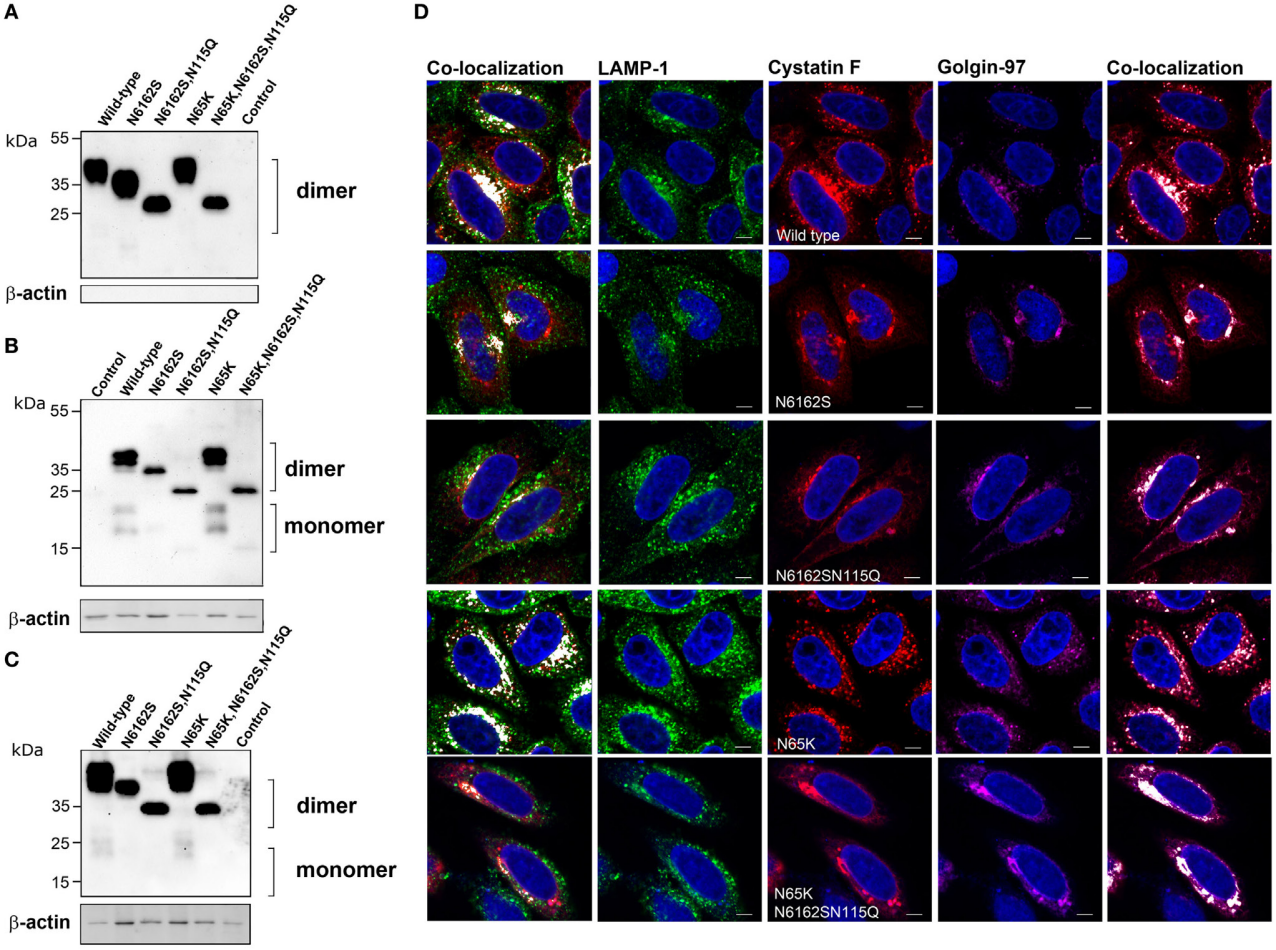
Subcellular localization, secretion and uptake of full-length cystatin F mutants. The different full-length cystatin F mutants were transfected into HeLa cells using pcDNA3 vector. HeLa cells transfected with empty pcDNA3 vector served as controls. To follow the uptake, cell culture medium containing different mutant forms of full-length cystatin F was added to non-transfected HeLa cells. Cell culture medium **(A)** and cell lysates of transfected **(B)** and recipient **(C)** cells were analyzed 24 h after transfection **(A,B)** or conditioning **(C)** by western blot, using non-reducing conditions. β-actin staining was used to show equivalent protein loading. **(D)** Cells were studied by immunofluorescence microscopy, following transfection of HeLa cells with different mutant forms of cystatin F. Cells were co-stained with lysosomal (LAMP-1) marker (red) anti-cystatin F antibody (green) and anti-human Golgin-97 (purple) antibody, followed by secondary antibodies conjugated with Alexa Fluor 488 (LAMP-1), Alexa Fluor 555 (cystatin F) and Alexa Fluor 633 (Golgin-97). Images were taken at 63× magnification.

The subcellular localization and trafficking of wild-type cystatin F and its mutants were analyzed in HeLa (Figure 2) and Hek293 (Figure S1 in Supplementary Material) cells that do not express endogenous cystatin F (14, 20). Transfection of the cells with wild-type cystatin F (wt) and a mutant with an altered legumain binding site (N65K) resulted in significant secretion of the protein into the culture medium (Figure 2A; Figure S1A in Supplementary Material). In accordance with previous reports (16, 19), both forms were present in the medium exclusively as dimers. Significant amounts of wild-type cystatin F and N65K mutant were retained within the transfected cells, both being only partially converted to the monomeric cystatin F (Figure 2B; Figure S1B in Supplementary Material). As shown by immunofluorescence microscopy, the intracellular fraction of wt cystatin F and N65K mutant was colocalized with “dot like” structures of endo-lysosomal components (LAMP-1 and Lysotracker Red DND labeling) and with components of the Golgi apparatus (Golgin-97 labeling) (Figure 2D; Figure S1D in Supplementary Material). Wild-type cystatin F and its N65K mutant, when added to the culture medium, were also taken effectively up by the cells and converted intracellularly to their monomeric forms (Figure 2C; Figure S1C in Supplementary Material).

Double (N6162S) unglycosylated and (N6162SN115Q) non-glycosylated mutants were also secreted and found as dimers in the cell culture medium. Both mutants were partially retained within the transfected cells (Figure 2B; Figure S1 in Supplementary Material) and were colocalized predominantly with the marker of the biosynthetic/secretory pathway (Golgin-97) (Figure 2D; Figure S1 in Supplementary Material). However, a small amount of their intracellular fraction can be seen as a monomeric form, suggesting M6P-independent trafficking of cystatin F to the endocytic pathway. Similarly, both double and fully unglycosylated mutants were detected in the recipient cells, although to a much lesser extent than cystatin F, revealing an additional, M6P-independent mechanism for cystatin F uptake (Figure 2C; Figure S1 in Supplementary Material). However, only a small fraction of the double unglycosylated mutant was present as monomer, and the monomeric form of the non-glycosylated mutant was absent in cell lysates.

### Subcellular Localization, Secretion, and Uptake of N-Terminally Truncated Cystatin F Mutants

**Figure 3:**
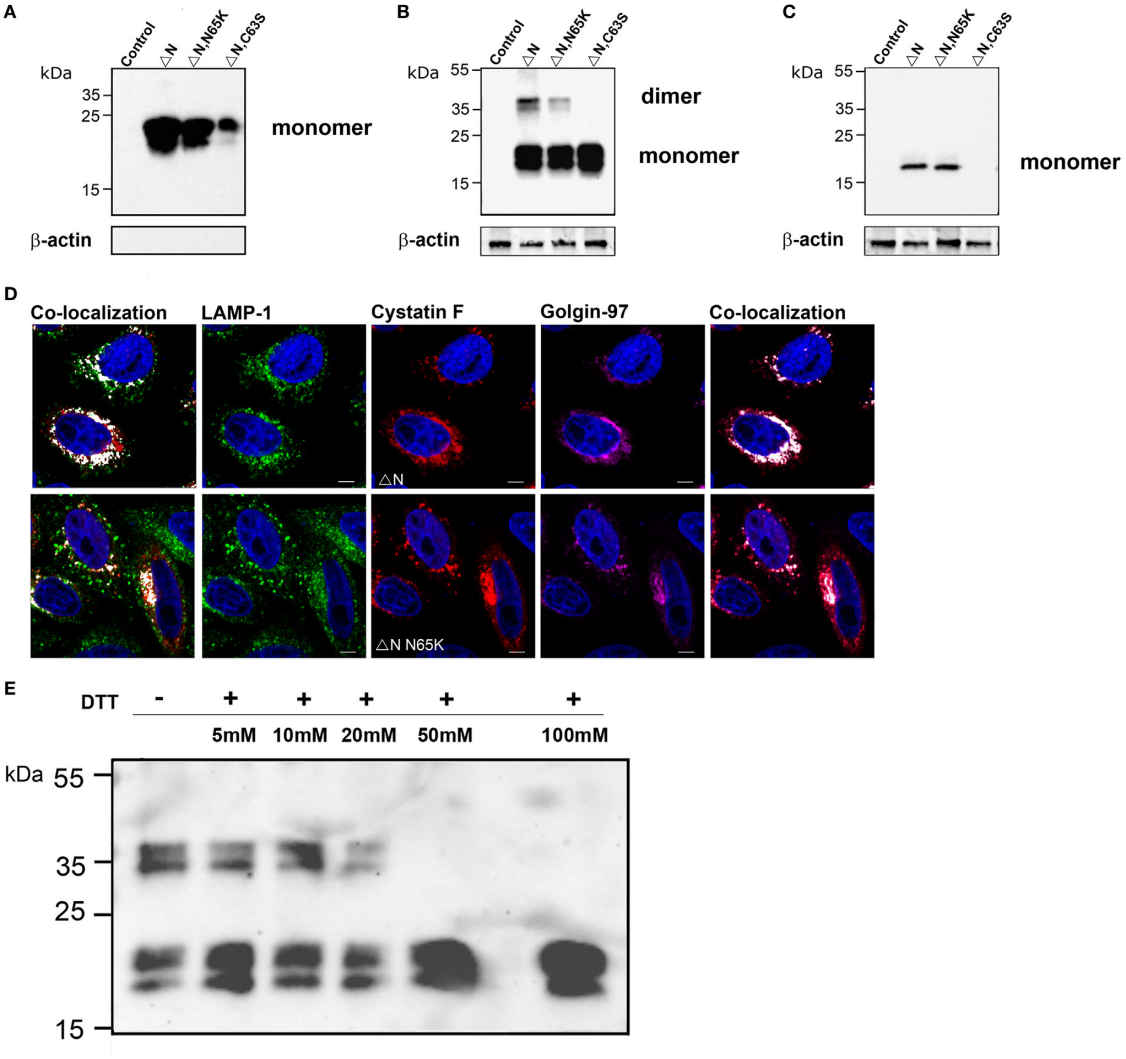
Subcellular localization, secretion, and uptake of N-terminally truncated cystatin F mutants. The N-terminally truncated cystatin F mutants were transfected into HeLa cells using pcDNA3 vector. HeLa cells transfected with empty pcDNA3 vector served as controls To follow the uptake, cell culture medium containing N-terminally truncated mutant forms of cystatin F was added to non-transfected HeLa cells (conditioning). Cell culture medium **(A)** and cell lysates of transfected **(B)** and recipient **(C)** cells were analyzed 24 h after transfection by western blot using non-reducing conditions. β-actin staining was used to show equivalent protein loading. **(D)** Following transfection of HeLa cells with different mutant forms of cystatin F, cells were co-stained with lysosomal (LAMP-1) marker (red) anti-cystatin F antibody (green) and anti-human Golgin-97 (purple) antibody and secondary antibodies conjugated with Alexa Fluor 488 (LAMP-1), and studied by immunofluorescence microscopy. Alexa Fluor 555 (Cystatin F) and Alexa Fluor 633 (Golgin-97). Images were taken at 63× magnification. **(E)** Cell lysates of N-terminally truncated cystatin F mutant were run under non-reducing and under different reducing conditions [5–100 mM dithiothreitol (DTT)] in SDS-PAGE and immunoblotted for cystatin F.

N-terminally truncated cystatin F (ΔN) and N-terminally truncated cystatin F N65K mutant (ΔN N65K) were secreted from transfected cells and were present in the medium solely in the monomeric form (Figure 3A). Surprisingly, a significant amount of these mutants was retained in transfected cells in both monomeric and dimeric forms (Figure 3B). Addition of DTT to cell lysates at concentrations higher than 25 mM, prior to SDS-PAGE analysis, reduced the dimeric form to monomers (Figure 3E). Since these mutants lack Cys26, due to the N-terminal truncation, the remaining Cys63 apparently mediates ΔN cystatin F dimerization. Indeed, mutagenesis of Cys63 to Ser completely abolished dimerization of N-terminally truncated cystatin F (Figure 3B; Figure S2B in Supplementary Material). In transfected cells, both (wt and N65K) N-terminally truncated mutants were colocalized with biosynthetic/secretory (Golgin-97) and endo-lysosomes (LAMP-1 or Lysotracker Red DND) markers (Figure 3D; Figure S2D in Supplementary Material). N-terminally truncated cystatin F (wt and N65K) were also taken up by recipient cells in which only monomeric forms were detected. The non-dimerizing ΔNC63S mutant was secreted from transfected cells but was not detected inside recipient cells, possibly due to the lower rate of its internalization (Figure 3C; Figure S2C in Supplementary Material).

### The Effects of Cystatin F Mutants on the Activity of Cathepsins H, L, C, and Legumain

**Figure 4:**
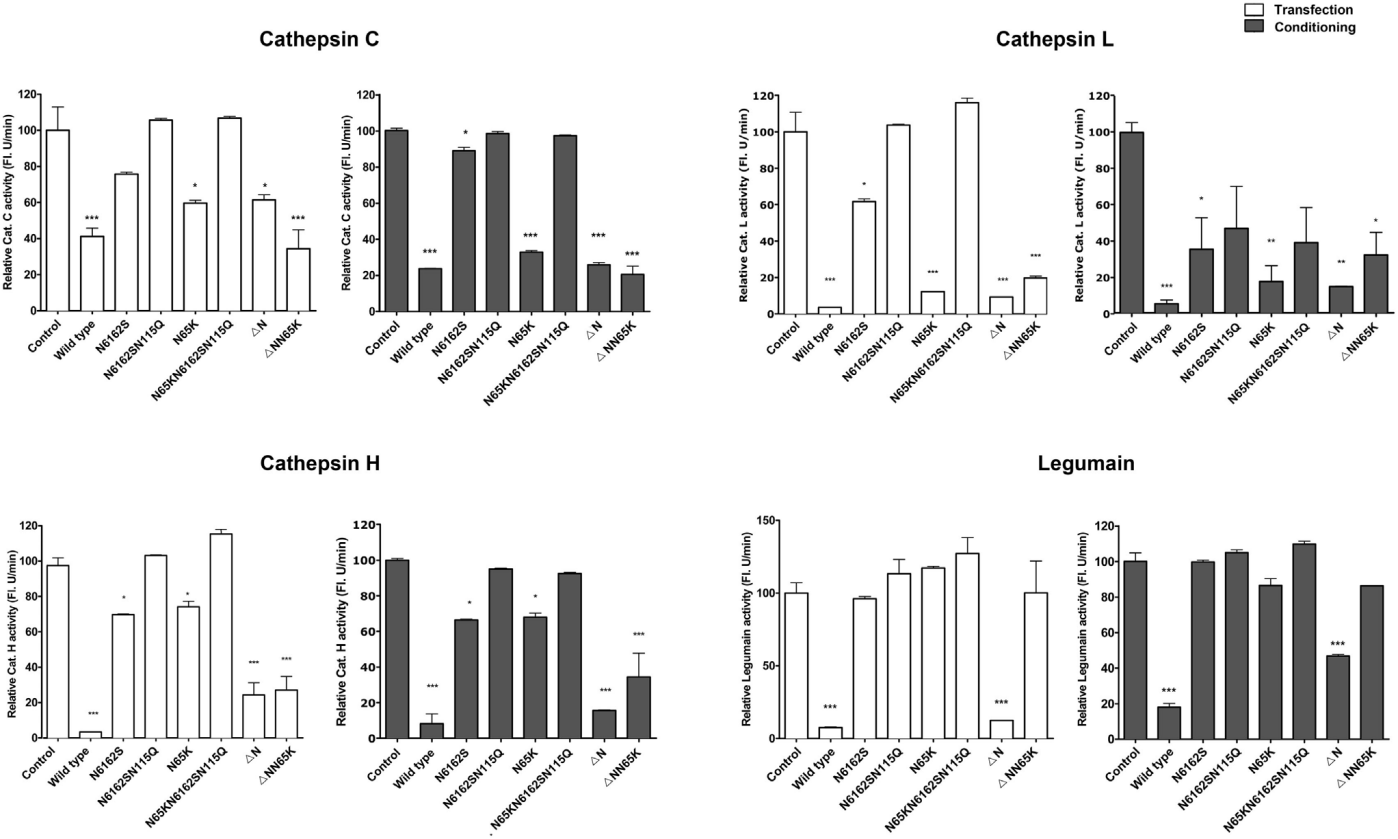
Effects of various cystatin F mutants on lysosomal protease activity. Cystatin F mutants were transfected into HeLa cells using pcDNA3 vector. HeLa cells transfected with empty pcDNA3 vector served as controls To follow the uptake, cell culture medium containing different mutant forms of cystatin F was added to non-transfected HeLa cells. Protease activity was measured in post-nuclear cell lysates prepared 24 h after the transfection (white columns) or after conditioning (exposure to conditioned cell culture medium—gray columns). Error bars represent SD of three replicates. Statistic indicators: *P ≤ 0.05; **P ≤ 0.01; ***P ≤ 0.001.

The effects of cystatin F mutants were examined on the activity of the intracellular peptidases cathepsins C, H, L and legumain in whole cell lysates prepared from cells that were either transfected (Figure 4) or exposed to cell culture medium containing cystatin F mutants for 24 h (Figure 4). The inhibition results from their trafficking to endosomal/lysosomal vesicles and monomerisation of cystatin F dimer. Wild-type cystatin F and N65K mutant reduced the activity of cathepsins L, H, and C in all cell lysates tested; however, the effect was weaker for cathepsin H than for the other two cathepsins. As expected, wt cystatin F inhibited legumain, while N65K mutants did not. Accordingly, N-terminally truncated cystatin F and ΔNN65K mutant both inhibited cathepsins L, C, and H, since they were already present in monomeric forms and did not need prior activation.

For the double unglycosylated mutant N6162S, the inhibition of cysteine cathepsins and legumain was much weaker than that of the fully glycosylated forms and wt cystatin F, in line with limited trafficking to the endosomal/lysosomal vesicles, Further, in accord with the absence of the monomeric form and the lack of localization to endo/lysosomal vesicles, the non-glycosylated cystatin F mutant (N6162SN115Q) did not inhibit any of the peptidases tested.

### The Effects of Cystatin F Mutants on the Cytotoxicity of NK-92 Cells and Primary NK Cells

**Figure 5:**
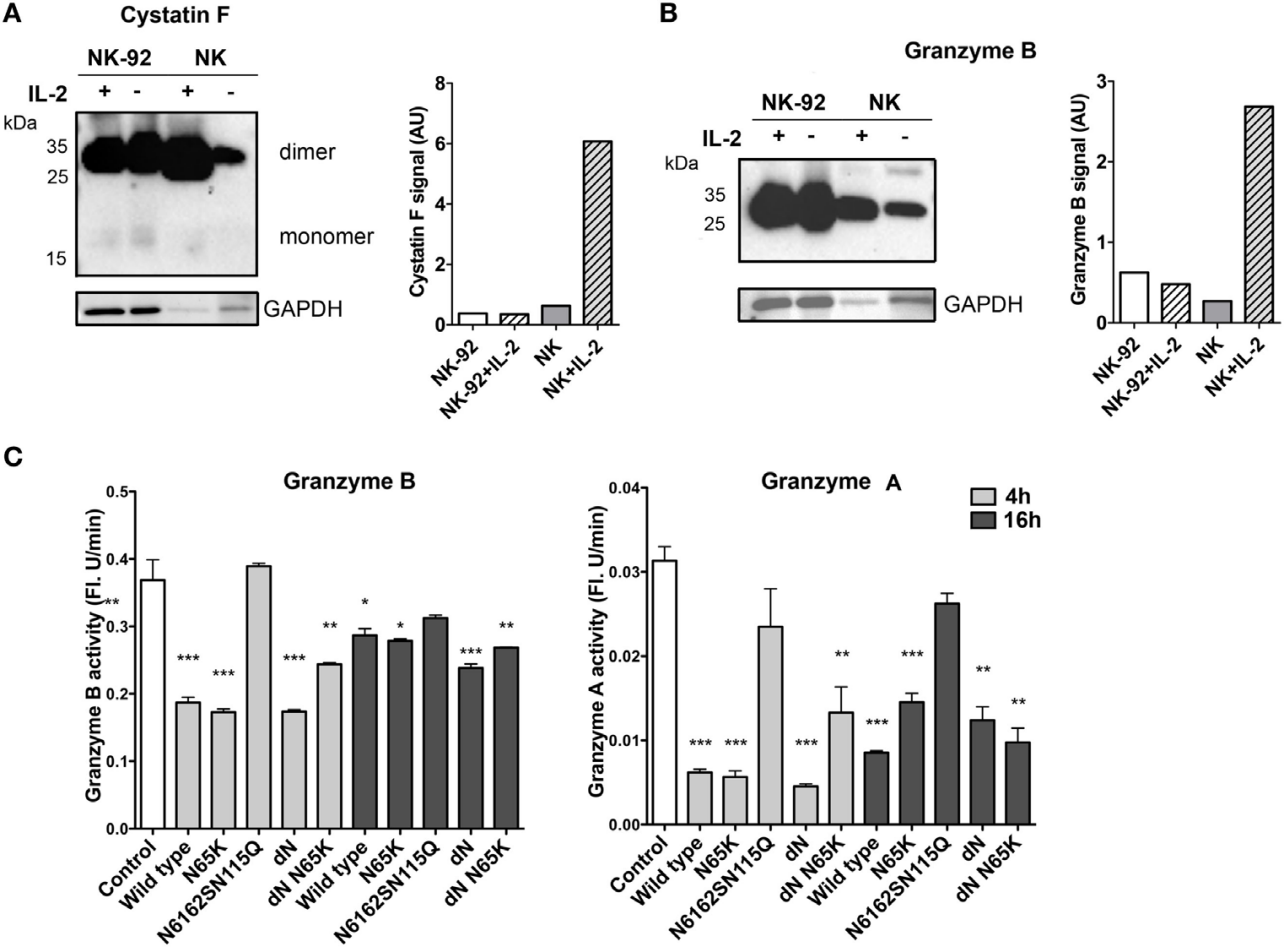
Expression and activity of the main components of cytotoxic machinery of NK-92 and isolated primary NK cells. Non-stimulated and IL-2-stimulated NK-92 and isolated primary NK cells were lysed and analyzed by non-reducing PAGE for protein levels of cystatin F **(A)** and granzyme B **(B)** by immunoblotting. Proteolytic activity of granzyme B and granzyme A was measured in post-nuclear cell lysates prepared from IL-2-stimulated NK-92 after 4 and 16 h incubation with different cystatin F mutants. Error bars represent SD of three replicates. Statistic indicators: *P ≤ 0.05; **P ≤ 0.01; ***P ≤ 0.001. **(C)**. GAPDH staining was used to show protein loading and signal intensities for cystatin F and granzyme B were normalized to the signal from loading control **(A,B)**.

In order to gain a better insight into the mechanisms involving cystatin F in its cytotoxic function, we determined its levels together with the activities of legumain, granzymes A and B and pro-granzyme convertases cathepsins C and H upon the stimulation of NK-92 and primary NK cells with IL-2, which is known to increase the expression of multiple effector molecules essential for NK cytotoxic function (45, 46). We showed that, on stimulation with IL-2, the expression of the dimeric form of cystatin F remained unchanged in NK-92 cells whereas, in primary NK cells, it was significantly increased (Figure 5A), similarly to that observed by Magister et al. (38). Again, the level of monomeric cystatin F remained unchanged in primary NK cells on IL-2 stimulation. Furthermore, the activity of cathepsin C increased in primary NK cells but not in NK-92 cells (Figure S5 in Supplementary Material). Similarly, unlike in NK-92 IL-2 significantly increased the protein level of granzyme B and activity levels of granzymes B and A in primary NK cells (Figure 5B; Figure S5C in Supplementary Material). To demonstrate that active monomeric cystatin F regulates the activation of granzymes A and B, we exposed NK-92 cell lysates to the same concentration (80 nM) of different cystatin F mutants. A significant decrease was observed in the activity of granzymes at both incubation times tested (Figure 5C), the effect being more pronounced after 4 h incubation. As expected, the ΔN mutant, present predominantly as an active monomer, caused the most significant decrease in the granzymes’ activities.

**Figure 6:**
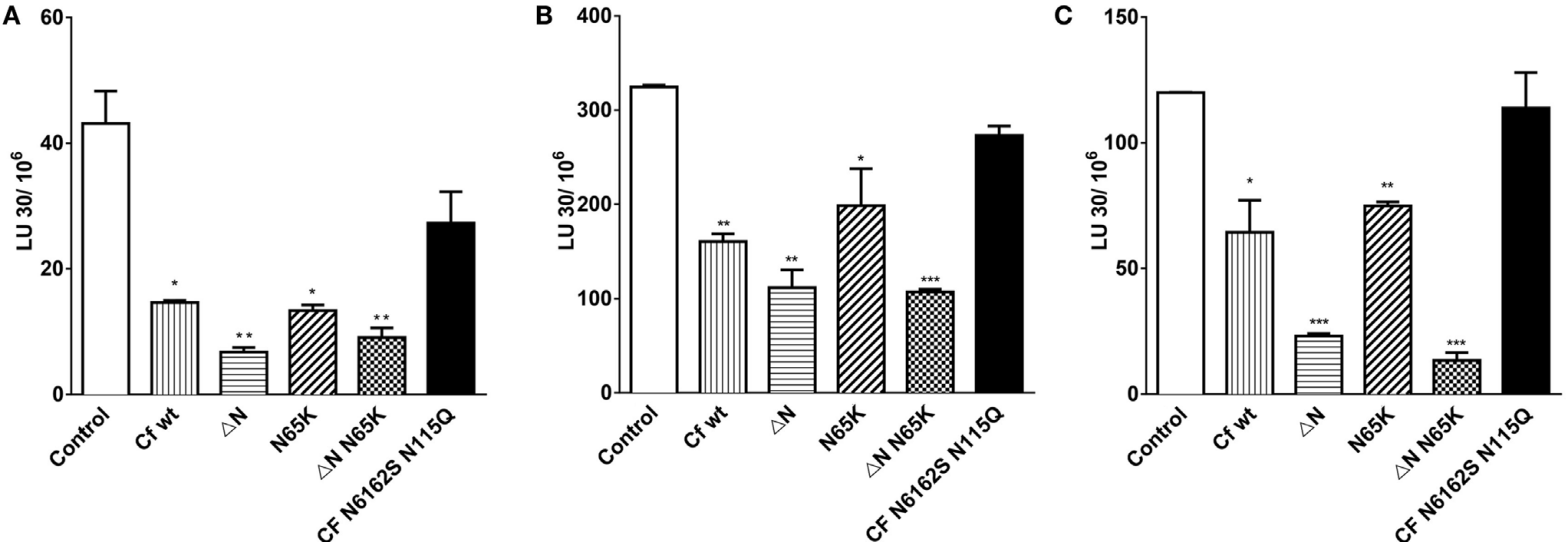
The effects of different mutant forms of cystatin F on the cytotoxicity of NK-92 and primary NK cells toward K562 target cells. Cytolytic activity of IL-2 activated NK-92 cells against K562 erythroleukemia cells at different target to effector ratios **(A)**. Cytolytic activities of primary NK cells isolated from two representative (healthy) individuals were cultured for 48 h with IL-2, and tested against K562 erythroleukemia cells at different target to effector ratios **(B,C)**. Various cystatin F mutants (80 nM) were added to effector and target mixtures and incubated for 4 h. % Cytotoxicity was determined at different E:T ratio, and LU 30/10^6^ cells were calculated using the inverse of the number of effectors needed to lyse 30% of the tumor cells × 100. Statistic indicators: *P ≤ 0.05, **P ≤ 0.01, and ***P ≤ 0.001.

Further, the effects of cystatin F mutants were determined on the granule-mediated cytotoxicity of NK-92 (Figure 6A) and of primary NK cells (Figures 6B,C) on stimulation with IL-2. To ensure that the NK cells used in our experiments kill target cells predominantly *via* the Ca^2+^-dependant granule release pathway, and not through Fas-mediated cell death, K562 erythroleukemia cells were chosen as target cells (47). Further, we demonstrated that primary NK cells are also capable of lysing MCF-7 cells, which have low levels of Fas receptor (FasR) and are resistant to anti-FasR antibody mediated apoptosis (48) (Figure S4 in Supplementary Material). As perforin activity is calcium dependent (49), the killing assay was performed in the presence of the calcium chelator EGTA, and MgCl_2_ was used to confirm that primary NK cells kill targets in the granule dependant pathway (Figure S4 in Supplementary Material). We showed that the incubation with wild-type cystatin F and its N-terminally truncated mutant F did not affect the lytic granule exocytosis in activated NK-92 cells (Figure S6 in Supplementary Material).

Wild-type cystatin F and its N65K mutant significantly decreased the cytotoxicity of NK-92 and primary NK cells (Figure 6; Figure S6 in Supplementary Material) at all target to effector ratios measured (*P* ≤ 0.05; *P* ≤ 0.01) whereas the wild-type cystatin F, incubated with the 2.5 M excess of the anti-cystatin F antibody did not affect the cytotoxicity of NK-92 cells (Figure S6 in Supplementary Material). Antibody alone also did not affect cell cytotoxicity. N-terminally truncated mutants of cystatin F had the most pronounced impact on cell cytotoxicity, reducing target cell killing by ≥20% in both NK-92 cells (*P* ≤ 0.01) and primary NK cells (*P* ≤ 0.01; *P* ≤ 0.001). Finally, the non-glycosylated mutant N6162SN115Q had no significant effect on cytotoxic killing of targets in either NK cell type tested.

## Discussion

Cystatin F has recently been identified as an upstream regulator of split anergy in NK cells (38). Since this inhibitor can be produced and secreted by tumor cells (34, 35), we investigated whether extracellular cystatin F can reduce the cytotoxicity of NK cells. We found that cystatin F, in both inactive dimeric and activated N-terminally truncated forms, can be taken up effectively by cells and targeted to lysosomes. Within the lysosomes, cystatin F inactivates cathepsins C and H, major convertases capable of generating active granzymes and the main executors of cell cytotoxicity. This, in turn, leads to diminished cytotoxicity. N-glycosylation of cystatin F has a significant impact on internalization. For mutants lacking two or three N-glycosylated sites, internalization gradually decreased. On the other hand, the binding site for C13 protease, legumain, was found to have no effect on cystatin F internalization or endosomal/lysosomal targeting. These results, therefore, offer an insight into the physiological consequences of the acquisition of cystatin F by bystander cells and its possible role as a mediator of immune suppression.

It has been shown that the regulatory function of cystatin F depends on two events—trafficking to the endosomal/lysosomal pathway and proteolytic cleavage of an N-terminal peptide (18, 20). The intracellular trafficking is critically dependent on the number of N-linked carbohydrates (16, 18). This is emphasized by the finding that, apart from canonical glycosylation sequences including Asn62 and Asn115, cystatin F possesses an alternative non canonical glycosylation site at Asn61 that can be glycosylated in the case of altered Asn62 (16). It was proposed that N-linked glycans on either Asn61 or Asn62 are required for M6P-dependent intracellular trafficking to endosomes/lysosomes and for cell uptake, whereas glycosylation at Asn115 is not important. To test this hypothesis on our cell model, we prepared a double unglycosylated mutant N6162S and a non-glycosylated mutant N6162SN115Q. Compared to wild-type cystatin F or to mutants without an altered glycosylation pattern, their presence is indeed lower within the endosomal/lysosomal pathway; however, it is not completely absent. In particular, the double N6162S mutant is able to co-localize with the lysosomal marker LAMP-1 and partially inhibits the proteolytic activity of various lysosomal peptidases, suggesting that glycosylation of Asn115 also contributes to M6P targeting and/or that endosomal/lysosomal trafficking of cystatin F could be, in part, glycosylation independent.

The question arose as to whether the N-terminally truncated monomeric form of cystatin F can also be targeted to the endo/lysosomal pathway, can be secreted, or can be internalized by recipient cells. In HeLa and Hek293 cells, we demonstrated that the truncated (ΔN mutant) cystatin F was effectively targeted to the endo/lysosomes, inhibiting the examined cathepsins. Like wild-type dimeric cystatin F, the truncated form was secreted to the medium and, moreover, was internalized by both HeLa and Hek293 cells. The latter observation appears to contradict those of Colbert et al. (16) that indicate that only the dimeric form of cystatin F can be taken up by mouse L929 cells. This apparent discrepancy can be explained by the difference in cell types/species used, i.e., Hek293T as producers and mouse L929 as receivers of cystatin F, given that mouse cystatin F has only one N-linked carbohydrate chain at a site homologous to Asn62 whereas, in human cystatin F, Asn62 and Asn115 can carry carbohydrate chains. The other explanation is that, despite the absence of Cys26, truncated cystatin F can be present in lysates of transfected cells as both a monomer and a dimer. The dimer dissociated only on exposure to reducing conditions (25 mM DTT) that are quite strong, although lower than those needed for dissociation of regular full-length cystatin F dimer [100 mM DTT (19)]. Similarly, Hamilton et al., showed dimer formation on substitution of Cys26 in full-length cystatin F (20). We investigated whether this dimeric form constitutes an intermediate that can enable translocation of cystatin F from biosynthetic cellular compartments or cell culture medium to endo/lysosomes. Dimerization was prevented by replacing Cys63 with Ser, confirming that the disulfide bond between Cys63 residues is responsible for dimerization of N-terminally truncated cystatin F. Therefore, besides the regular cystatin F dimer, formed through two disulfide bonds between Cys26 on one subunit and Cys63 on the other, we can find, in extracellular space, a dimer of truncated, activated cystatin F, formed by a disulfide bond between two Cys63 residues. Active monomeric cystatin F, derived from ΔN cystatin F dimer, could inhibit the extracellularly located cysteine peptidases. This is important for cancer cells, particularly those at the invasive edges of tumors, as they often shift the lysosomes from a perinuclear area to the cell periphery, secreting the lysosomal contents into the extracellular space (50). Furthermore, these results extend the concept of “in trans” regulation of the peptidases to activated truncated cystatin F which, upon internalization to recipient cells, does not require the presence of an activating peptidase, such as the putative activator cathepsin V (51), for monomerization and subsequent regulation of lysosomal peptidases.

The second binding site of cystatin F, inhibiting the C13 peptidase family, could also affect cell trafficking and internalization. To test this possibility, we prepared a full-length and an N-terminally truncated N65K mutant, which is not able to bind legumain, a member of C13 peptidases. In HeLa and Hek293 cells, which do not normally express cystatin F (14, 20), a transfected N65K mutant was targeted to endo/lysosomes to the same extent as the wild-type cystatin F. Both forms were taken up by the cells and converted to their active forms, indicating that the binding site for C13 proteases is not involved in the trafficking, internalization, or activation of cystatin F. The ability to inhibit cathepsins C and L did not differ significantly between these two forms; however, the effect of the N65K mutant on the activity of cathepsin H was less pronounced, probably because legumain is a peptidase involved in the activation of cathepsin H (52).

Its unique expression pattern and inhibitory properties make cystatin F a good candidate for the regulation of granule-mediated cell cytotoxicity. It has been shown that, in NK cells, cystatin F is present, not only in the dimeric form but in an N-terminally truncated monomeric form that potently inhibits granzyme convertase cathepsin C (20, 38). Furthermore, in human cytotoxic CD8+ T cells, which share the same granule-mediated cytotoxic mechanism as NK cells, the interaction of cystatin F with cathepsin C was confirmed by immmunoprecipitation (20).

The impact of cystatin F on the cytotoxicity of NK cells was studied using the NK-92 cell line and primary NK cells isolated from peripheral blood, both stimulated with IL-2. The stimulation of NK cells with IL-2 results in activation of a set of genes coding for effector molecules (perforin, granzyme B, Fas ligand, and TRAIL) which increase NK cell lytic potential. However, as in the previous study (38), the IL-2 stimulation increased the cytotoxicity of primary NK cells more significantly than that of NK-92 cells (Figure 5). In line with this was the increased activity of cathepsin C and protein and the activity levels of granzyme B in primary NK cells, but not in NK-92 cells. Cystatin F was also significantly increased in primary NK cells after stimulation with IL-2, but only as a dimer—the level of the monomeric form remained unchanged. The increase in transcription and *de novo* synthesis of granzymes (45, 46), together with the zymogen activation of cathepsin C and the unchanged level of monomeric active cystatin F, therefore correlates with the increased cytotoxicity of primary NK cells upon stimulation with IL-2. It is not clear why the increased dimeric cystatin F is not processed into active monomers. Maybe, dimers do not reach the endosomal/lysosomal vesicles or IL-2 does not stimulate the expression of activating protease. However, the addition of cystatin F wt and its mutants to IL-2-stimulated primary NK cells and to NK-92 cells led to a significant decrease in their cytotoxicity toward K562 targets. As expected, the effect was more pronounced with active monomeric mutants, which effectively reduced cell cytotoxicity in both cell types. However, the decrease in cytotoxicity was significant, with wt cystatin F and full-length mutants forming inactive dimer, meaning that NK cells possess a peptidase that activates dimeric cystatin F within the endosomal/lysosomal vesicles. It has been reported that unstimulated NK-92 cells express cathepsin V (51) whereas there is no information on the expression of cathepsin V in activated NK-92 cells and in primary NK cells. Besides pro-granzyme convertases cathepsins C and H, active monomeric cystatin F can inhibit cathepsin L, a peptidase involved in processing perforin from its precursor form. Its lower level can, in addition, reduce NK cell cytotoxicity. The addition of fully unglycosylated cystatin F (non-glycosylated mutant N6162SN115Q) had no significant effects on the cytotoxicity of NK-92 and primary NK cells, confirming that, as in HeLa and Hek293 cells, non-glycosylated cystatin F is not internalized and targeted to lysosomes. Cystatin F, with a mutated binding site for legumain, showed the same effect on NK cell cytotoxicity as the wild-type cystatin F. This could be explained partially by the very low activity of legumain in NK-92 cells (51). IL-2 reduced legumain activity in primary NK cells although its level remained higher than in NK-92 cells (Figure S5 in Supplementary Material). Nevertheless, the role of legumain in the activation of effector molecules of cytotoxic cells remains controversial. It has been reported that legumain-null mice display lower NK cell activity (11). This could be a result of diminished perforin processing, since legumain has been shown to process cathepsin L (10). Alternatively, legumain inhibition should result in decreased activation of cathepsin H, and consequently pro-granzyme activation; however, our results do not support these observations.

## Conclusion

Our results demonstrate that cystatin F secreted from target cells can be internalized to NK cells and affect their cytotoxic effectiveness. The effect is most pronounced for dimeric inactive cystatin F; however, its N-terminally truncated, active monomeric form can also be internalized and enter the endo/lysosomal compartments. We have shown that the glycosylation pattern determines the internalization rate of both dimeric and monomeric cystatin F. Within the endosomal/lysosomal vesicles and secretory granules, active cystatin F decreases the activity of cathepsins C and H, pro-granzyme convertases and cathepsin L, involved in the processing of perforin, in this way regulating NK cell cytotoxicity. Increased expression and secretion of cystatin F in human cancer cell lines (34) and metastatic cells (35) have been presented, however clear *in vivo* evidence showing that cystatin F secreted from tumor cells or cells present in the tumor microenvironment can indeed affect the activity of NK cells has yet to be demonstrated. Prevention of cell internalization or monomerization of cystatin F by modifying the glycosylation profile or by targeting the peptidase responsible for cystatin F activation could greatly increase the cytotoxic potential of NK cells and improve the existing immunotherapies for cancer patients.

## Author Contributions

MPN, JS, and JK conceived and designed the experiments. MPN performed the experiments. JS contributed to the implementation of the research. UŠ provided help and material for some experiments. AJ provided ideas to design the study. MPN and JK wrote the manuscript. All authors reviewed the manuscript.

## Conflict of Interest Statement

The authors declare that the research was conducted in the absence of any commercial or financial relationships that could be construed as a potential conflict of interest.
