# Supplementary material for: Cystatin F Affects Natural Killer Cell Cytotoxicity
Source: Front Immunol. 2017 Nov 13;8:1459. doi: 10.3389/fimmu.2017.01459 (PMC5693851; doi:10.3389/fimmu.2017.01459)
Supplement: Supplementary file 1 [file Table_1.docx]

**Supplementary Table S1:** List of oligonucleotides used in cloning of different mutant forms of cystatin F. The sequence corresponding to the C-terminal portion of the signal peptide is double underlined in primers for ΔN mutant and substitutions and His-tag insertions are underlined in other primers

| ***cystatin F variants*** | ***Primer sequence (5’-3’)*** |
| --- | --- |
| **Wild type** | **cysF_upstream:** GTCAAGCTTATGCTGCCTGAGAAGG  **cysF_downstream**: GTCAAGCTTATGCGAGCGGCTGGA |
| **∆N** | **dNCysF-F:** GGTCTTGAGCACCACTGGGAAGCCAGGATTTCCTAAAACAAT  **dNCysF-R**: GTTTTAGGAAATCCTGGCTTCCCAGTGGTGCTCAAGACCAGGC |
| **∆N C63S** | **C63S CysF-F:** CATGTCGTTCGTGGAGTTGTTGAACTT  **C63S CysF-R**: AAGTTCAACAACTCCACGAACGACATG |
| **N6162S** | **cysF_N61-62S_F:** AGATACAGTGTTGAAAAGTTCAGCTCCTGCACGAACGACATGTTCTTGT  **cysF_N61-62S_R:** ACAAGAACATGTCGTTCGTGCAGGAGCTGAACTTTTCAACACTGTAT |
| **N6162S N115Q** | **CysF-N96Q:** TGTGACTTCCAAACCCAGCACACCTTGAAGCAG  **CysF-N96Qc:** CTGCTTCAAGGTGTGCTGGGTTTGGAAGTCACA |
| **N65K** | **CysF-N65K F:** GTTCAACAACTGCACGAAAGACATGTTCTTGTTCAAGGAGTC  **CysF- N65K R:** GACTCCTTGAACAAGAACATGTCTTTCGTGCAGTTGTTGAACTTTTC |
| **N6162S N65K N115Q** | **CysF-N6162SN65K F:** AAGTTCAGCTCCTGCACGAAAGACATGTTCTTGTTCAAGGAGTC  **CysF-N6162SN65K R:** GACTCCTTGAACAAGAACATGTCTTTCGTGCAGGAGCTGAACTTTTC |
| **∆N N65K** | **CysF-N65K F:** GTTCAACAACTGCACGAAAGACATGTTCTTGTTCAAGGAGTC  **CysF- N65K R:** GACTCCTTGAACAAGAACATGTCTTTCGTGCAGTTGTTGAACTTTTC |
| **His tagged**  **wt cystatin F** | **cysF-6H-pCDNA_F:** GTTCTCCGTTGTCACCATCACCATCACCATTGAGGATCCGAATTCT  **cysF-6H-pCDNA_R:** AGAATTCGGATCCTCAATGGTGATGGTGATGGTGACAACGGAGAAC |
| **His tagged**  **N6162S N115Q cystatin F** | **cysF-6H-pCDNA_F:** GTTCTCCGTTGTCACCATCACCATCACCATTGAGGATCCGAATTCT  **cysF-6H-pCDNA_R:** AGAATTCGGATCCTCAATGGTGATGGTGATGGTGACAACGGAGAAC |
| **His tagged**  **N65K cystatin F** | **cysF-6H-pCDNA_F:** GTTCTCCGTTGTCACCATCACCATCACCATTGAGGATCCGAATTCT  **cysF-6H-pCDNA_R:** AGAATTCGGATCCTCAATGGTGATGGTGATGGTGACAACGGAGAAC |
| **His tagged**  **ΔN cystatin F** | **cysF-6H-pCDNA_F:** GTTCTCCGTTGTCACCATCACCATCACCATTGAGGATCCGAATTCT  **cysF-6H-pCDNA_R:** AGAATTCGGATCCTCAATGGTGATGGTGATGGTGACAACGGAGAAC |
| **His tagged**  **ΔN N65K cystatin F** | **cysF-6H-pCDNA_F:** GTTCTCCGTTGTCACCATCACCATCACCATTGAGGATCCGAATTCT  **cysF-6H-pCDNA_R:** AGAATTCGGATCCTCAATGGTGATGGTGATGGTGACAACGGAGAAC |
