# Supplementary material for: Cystatin F Affects Natural Killer Cell Cytotoxicity
Source: Front Immunol. 2017 Nov 13;8:1459. doi: 10.3389/fimmu.2017.01459 (PMC5693851; doi:10.3389/fimmu.2017.01459)
Supplement: Supplementary file 2 [file Presentation_1.pptx]

## Slide 1
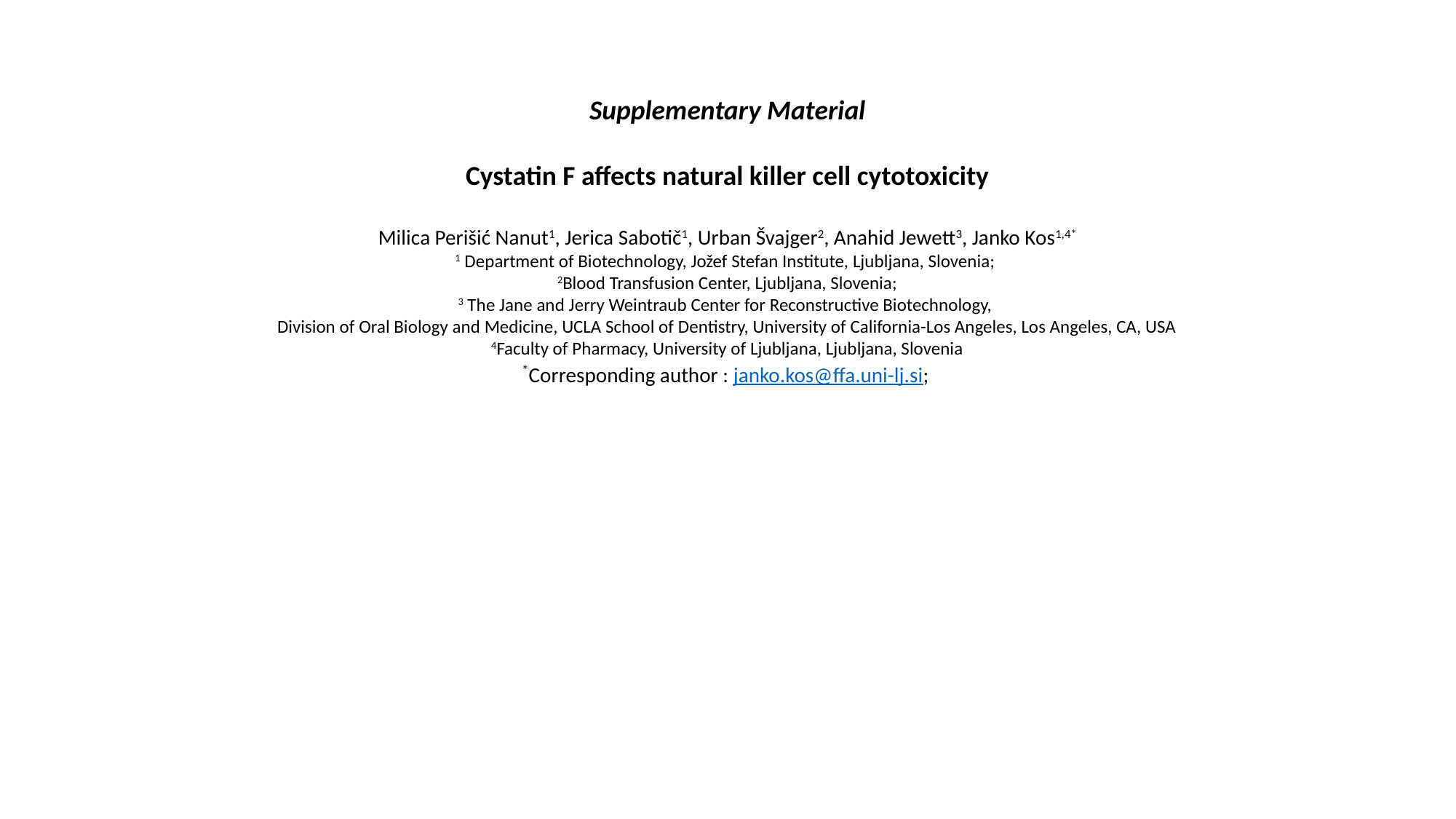

Supplementary Material
Cystatin F affects natural killer cell cytotoxicity
Milica Perišić Nanut1, Jerica Sabotič1, Urban Švajger2, Anahid Jewett3, Janko Kos1,4*
1 Department of Biotechnology, Jožef Stefan Institute, Ljubljana, Slovenia;
2Blood Transfusion Center, Ljubljana, Slovenia;
3 The Jane and Jerry Weintraub Center for Reconstructive Biotechnology,
Division of Oral Biology and Medicine, UCLA School of Dentistry, University of California-Los Angeles, Los Angeles, CA, USA
4Faculty of Pharmacy, University of Ljubljana, Ljubljana, Slovenia
*Corresponding author : janko.kos@ffa.uni-lj.si;

## Slide 2
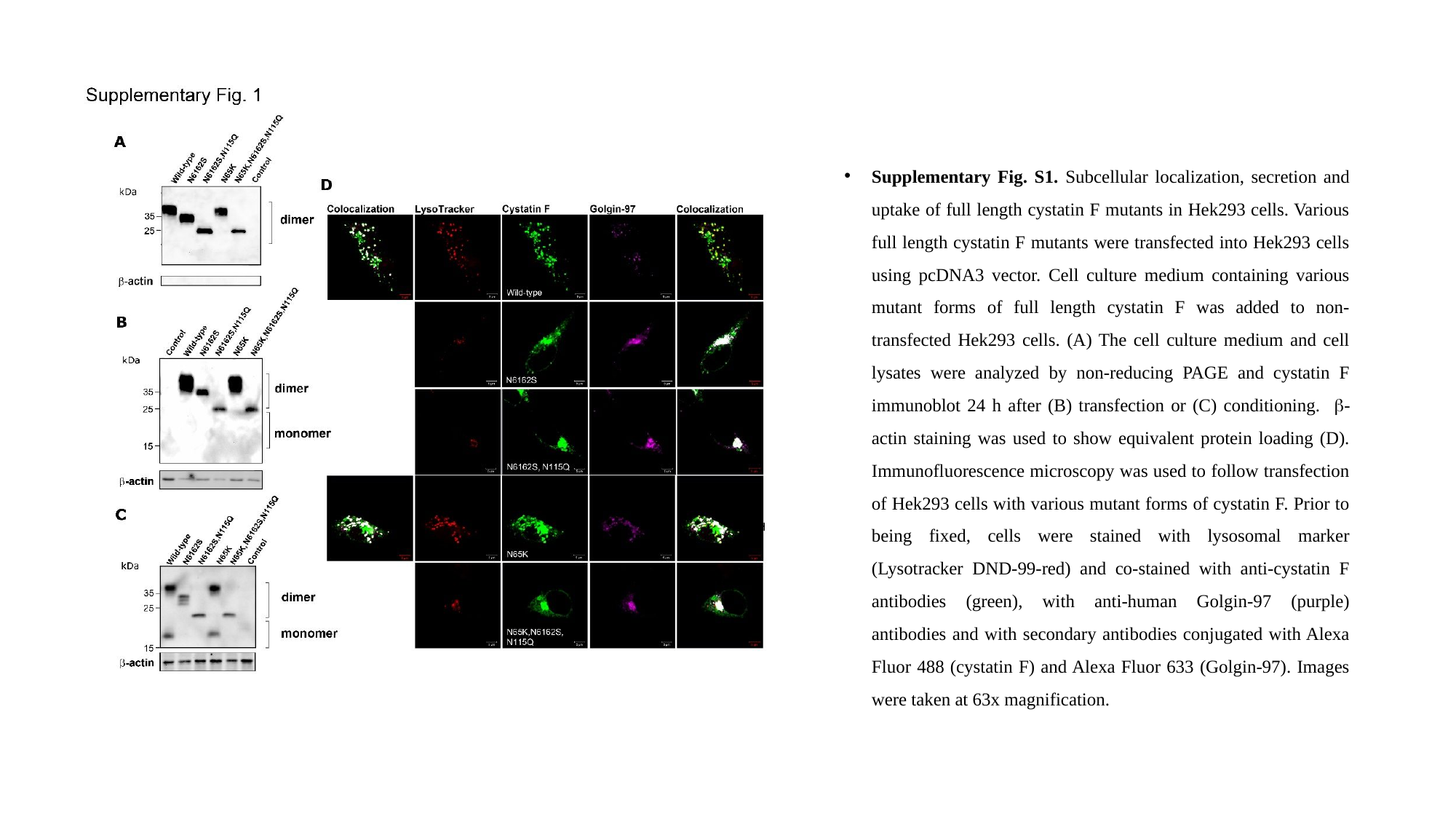

Supplementary Fig. S1. Subcellular localization, secretion and uptake of full length cystatin F mutants in Hek293 cells. Various full length cystatin F mutants were transfected into Hek293 cells using pcDNA3 vector. Cell culture medium containing various mutant forms of full length cystatin F was added to non-transfected Hek293 cells. (A) The cell culture medium and cell lysates were analyzed by non-reducing PAGE and cystatin F immunoblot 24 h after (B) transfection or (C) conditioning. b-actin staining was used to show equivalent protein loading (D). Immunofluorescence microscopy was used to follow transfection of Hek293 cells with various mutant forms of cystatin F. Prior to being fixed, cells were stained with lysosomal marker (Lysotracker DND-99-red) and co-stained with anti-cystatin F antibodies (green), with anti-human Golgin-97 (purple) antibodies and with secondary antibodies conjugated with Alexa Fluor 488 (cystatin F) and Alexa Fluor 633 (Golgin-97). Images were taken at 63x magnification.

## Slide 3
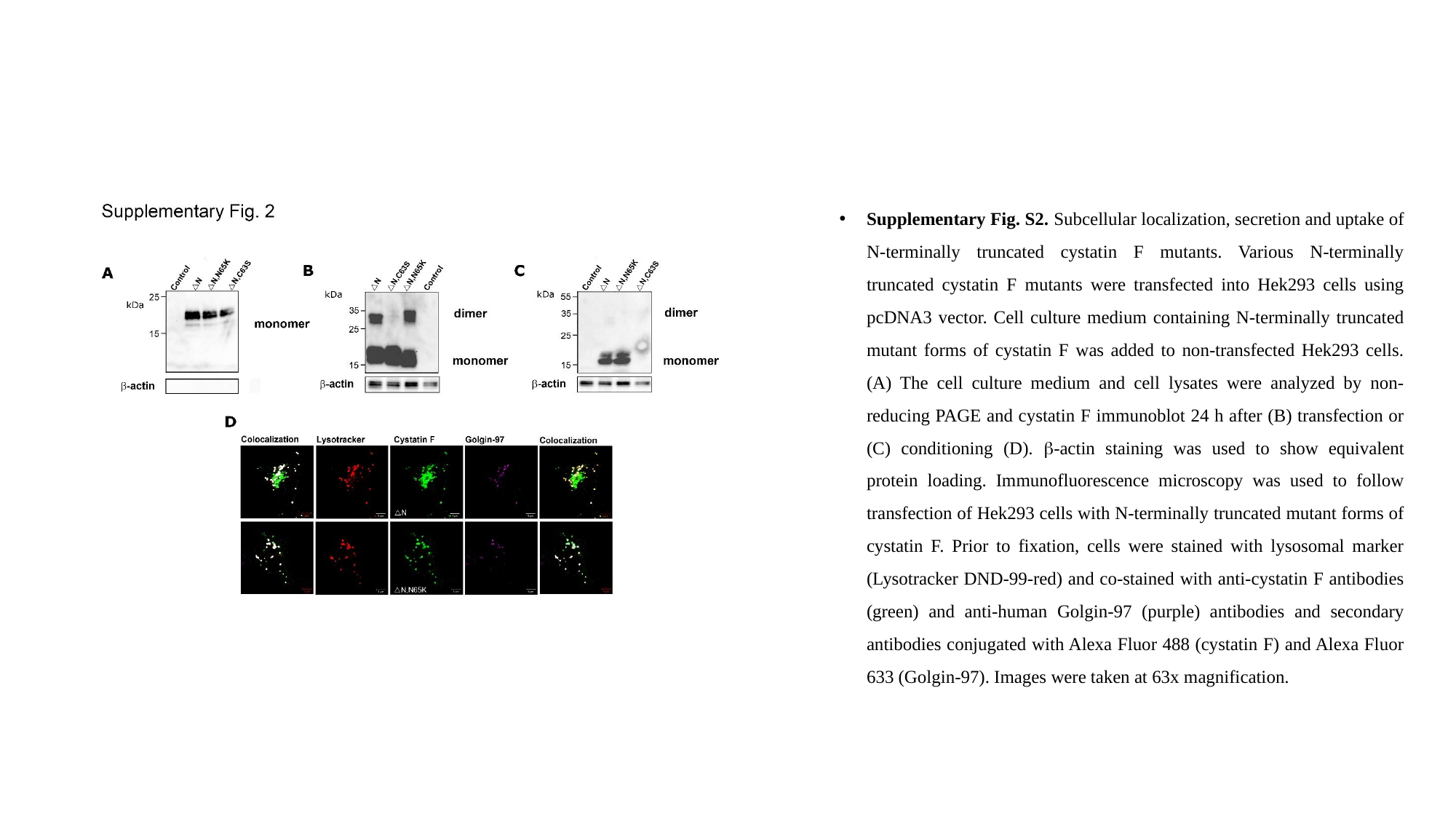

Supplementary Fig. S2. Subcellular localization, secretion and uptake of N-terminally truncated cystatin F mutants. Various N-terminally truncated cystatin F mutants were transfected into Hek293 cells using pcDNA3 vector. Cell culture medium containing N-terminally truncated mutant forms of cystatin F was added to non-transfected Hek293 cells. (A) The cell culture medium and cell lysates were analyzed by non-reducing PAGE and cystatin F immunoblot 24 h after (B) transfection or (C) conditioning (D). b-actin staining was used to show equivalent protein loading. Immunofluorescence microscopy was used to follow transfection of Hek293 cells with N-terminally truncated mutant forms of cystatin F. Prior to fixation, cells were stained with lysosomal marker (Lysotracker DND-99-red) and co-stained with anti-cystatin F antibodies (green) and anti-human Golgin-97 (purple) antibodies and secondary antibodies conjugated with Alexa Fluor 488 (cystatin F) and Alexa Fluor 633 (Golgin-97). Images were taken at 63x magnification.

## Slide 4
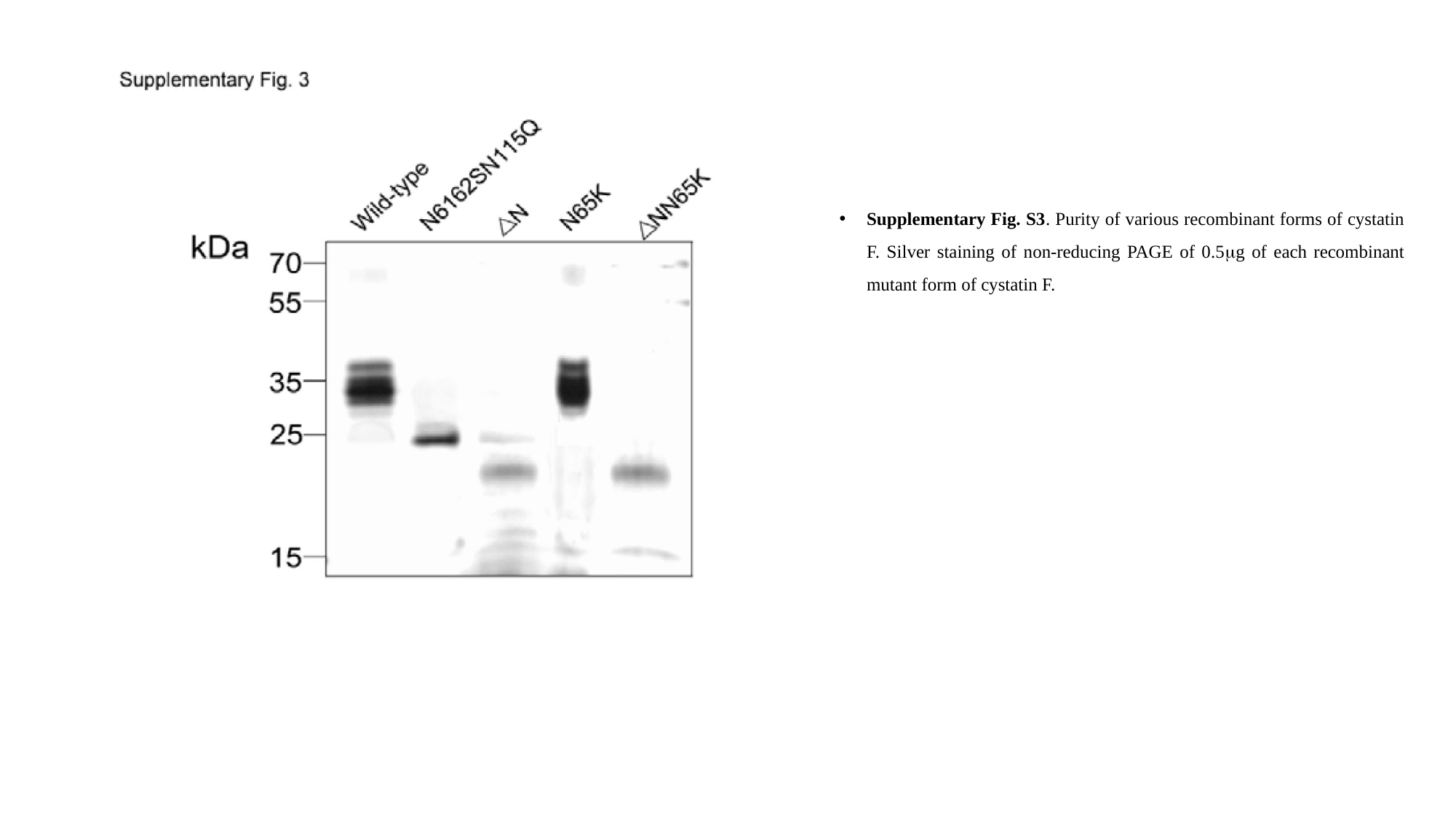

Supplementary Fig. S3. Purity of various recombinant forms of cystatin F. Silver staining of non-reducing PAGE of 0.5mg of each recombinant mutant form of cystatin F.

## Slide 5
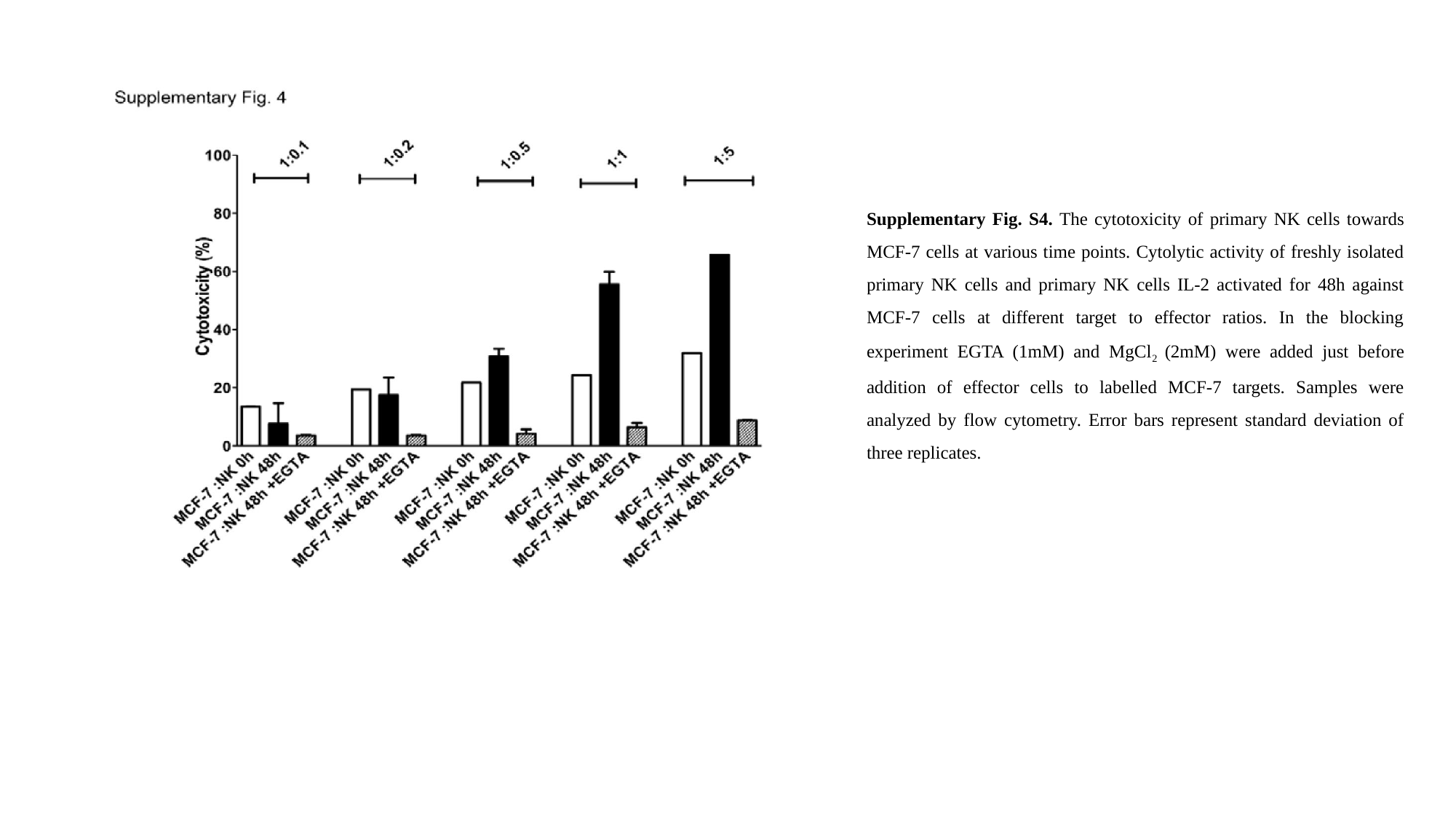

Supplementary Fig. S4. The cytotoxicity of primary NK cells towards MCF-7 cells at various time points. Cytolytic activity of freshly isolated primary NK cells and primary NK cells IL-2 activated for 48h against MCF-7 cells at different target to effector ratios. In the blocking experiment EGTA (1mM) and MgCl2 (2mM) were added just before addition of effector cells to labelled MCF-7 targets. Samples were analyzed by flow cytometry. Error bars represent standard deviation of three replicates.

## Slide 6
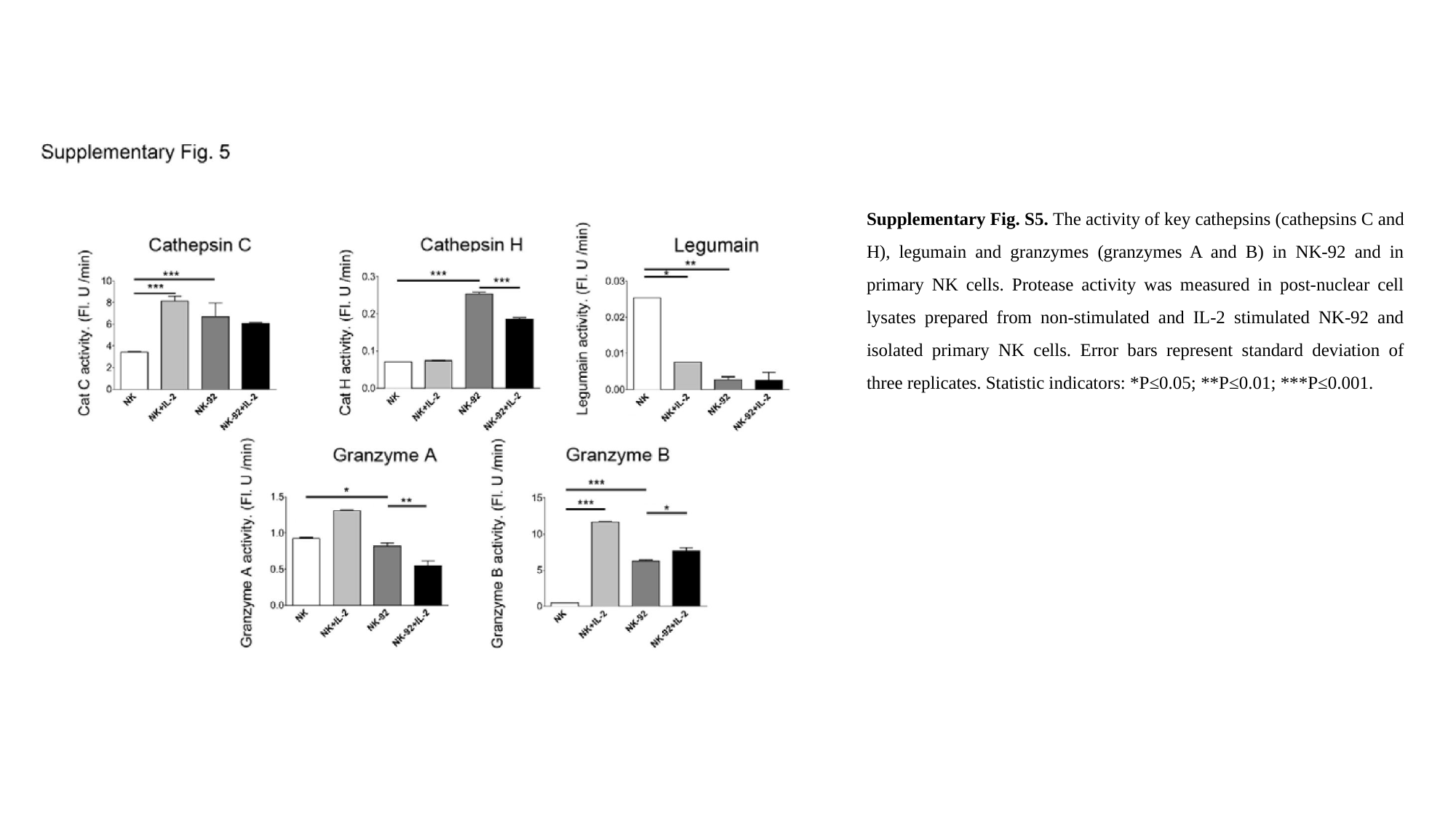

Supplementary Fig. S5. The activity of key cathepsins (cathepsins C and H), legumain and granzymes (granzymes A and B) in NK-92 and in primary NK cells. Protease activity was measured in post-nuclear cell lysates prepared from non-stimulated and IL-2 stimulated NK-92 and isolated primary NK cells. Error bars represent standard deviation of three replicates. Statistic indicators: *P≤0.05; **P≤0.01; ***P≤0.001.

## Slide 7
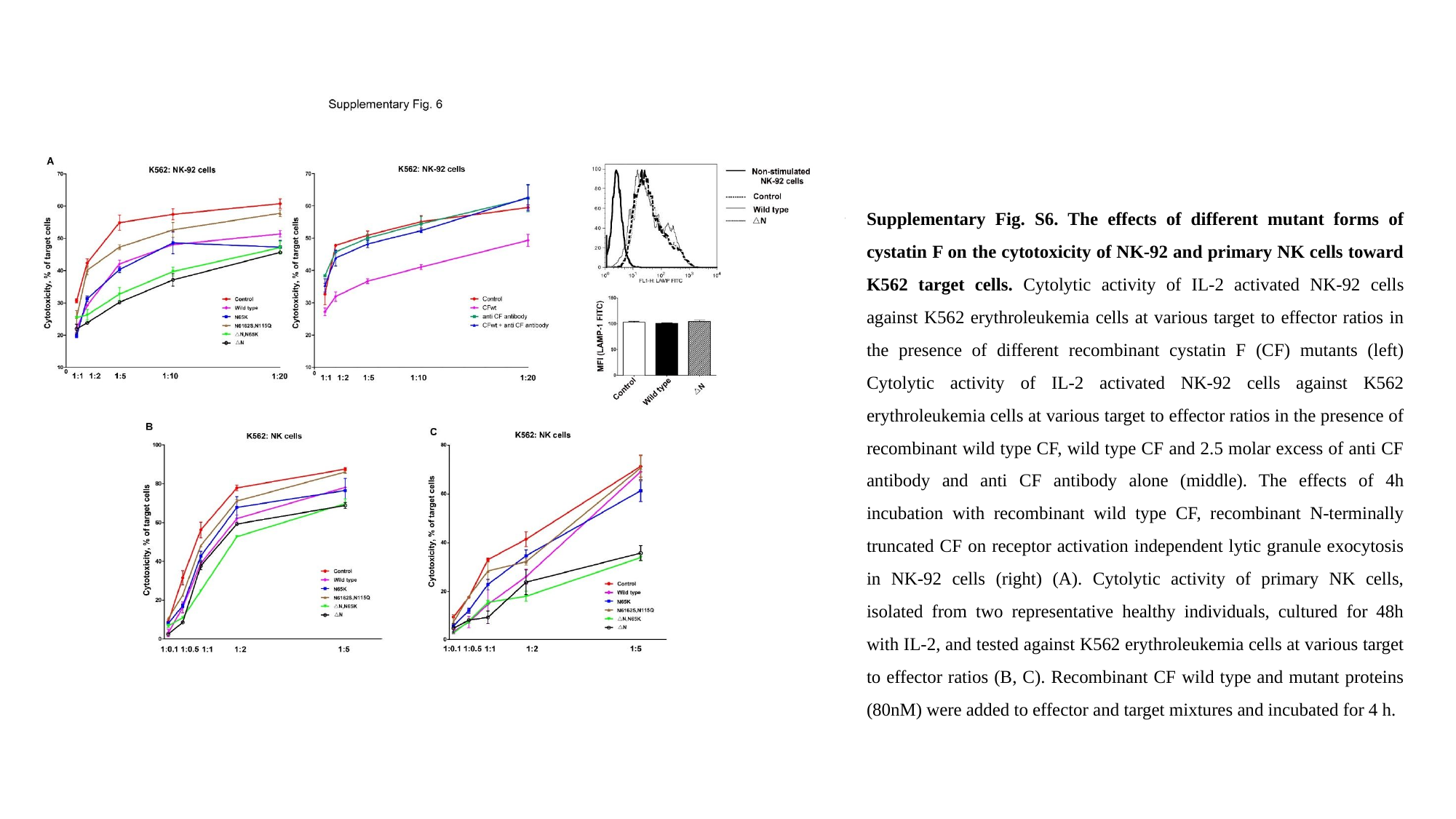

Supplementary Fig. S6. The effects of different mutant forms of cystatin F on the cytotoxicity of NK-92 and primary NK cells toward K562 target cells. Cytolytic activity of IL-2 activated NK-92 cells against K562 erythroleukemia cells at various target to effector ratios in the presence of different recombinant cystatin F (CF) mutants (left) Cytolytic activity of IL-2 activated NK-92 cells against K562 erythroleukemia cells at various target to effector ratios in the presence of recombinant wild type CF, wild type CF and 2.5 molar excess of anti CF antibody and anti CF antibody alone (middle). The effects of 4h incubation with recombinant wild type CF, recombinant N-terminally truncated CF on receptor activation independent lytic granule exocytosis in NK-92 cells (right) (A). Cytolytic activity of primary NK cells, isolated from two representative healthy individuals, cultured for 48h with IL-2, and tested against K562 erythroleukemia cells at various target to effector ratios (B, C). Recombinant CF wild type and mutant proteins (80nM) were added to effector and target mixtures and incubated for 4 h.

## Slide 8
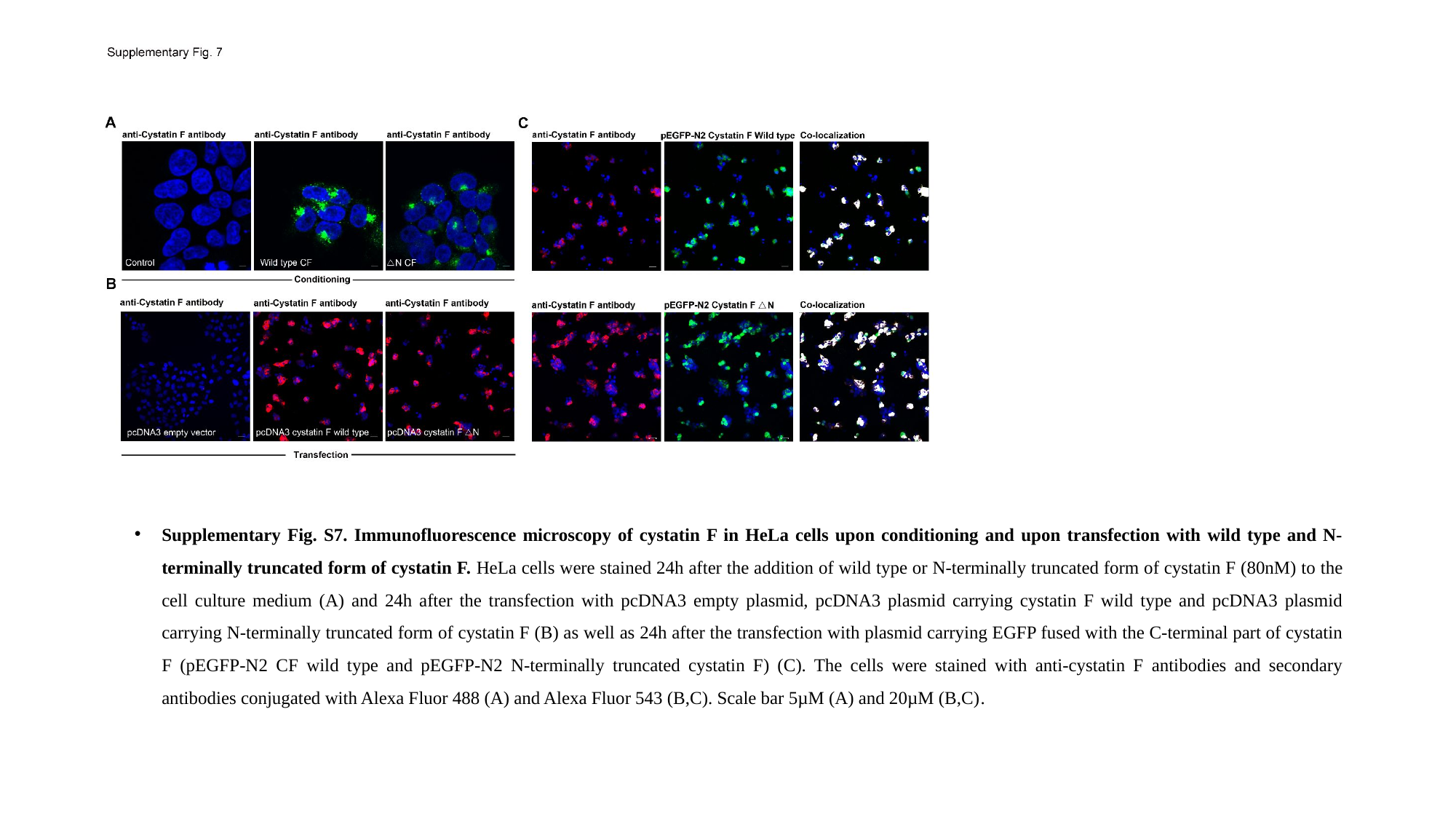

Supplementary Fig. S7. Immunofluorescence microscopy of cystatin F in HeLa cells upon conditioning and upon transfection with wild type and N-terminally truncated form of cystatin F. HeLa cells were stained 24h after the addition of wild type or N-terminally truncated form of cystatin F (80nM) to the cell culture medium (A) and 24h after the transfection with pcDNA3 empty plasmid, pcDNA3 plasmid carrying cystatin F wild type and pcDNA3 plasmid carrying N-terminally truncated form of cystatin F (B) as well as 24h after the transfection with plasmid carrying EGFP fused with the C-terminal part of cystatin F (pEGFP-N2 CF wild type and pEGFP-N2 N-terminally truncated cystatin F) (C). The cells were stained with anti-cystatin F antibodies and secondary antibodies conjugated with Alexa Fluor 488 (A) and Alexa Fluor 543 (B,C). Scale bar 5µM (A) and 20µM (B,C).

## Slide 9
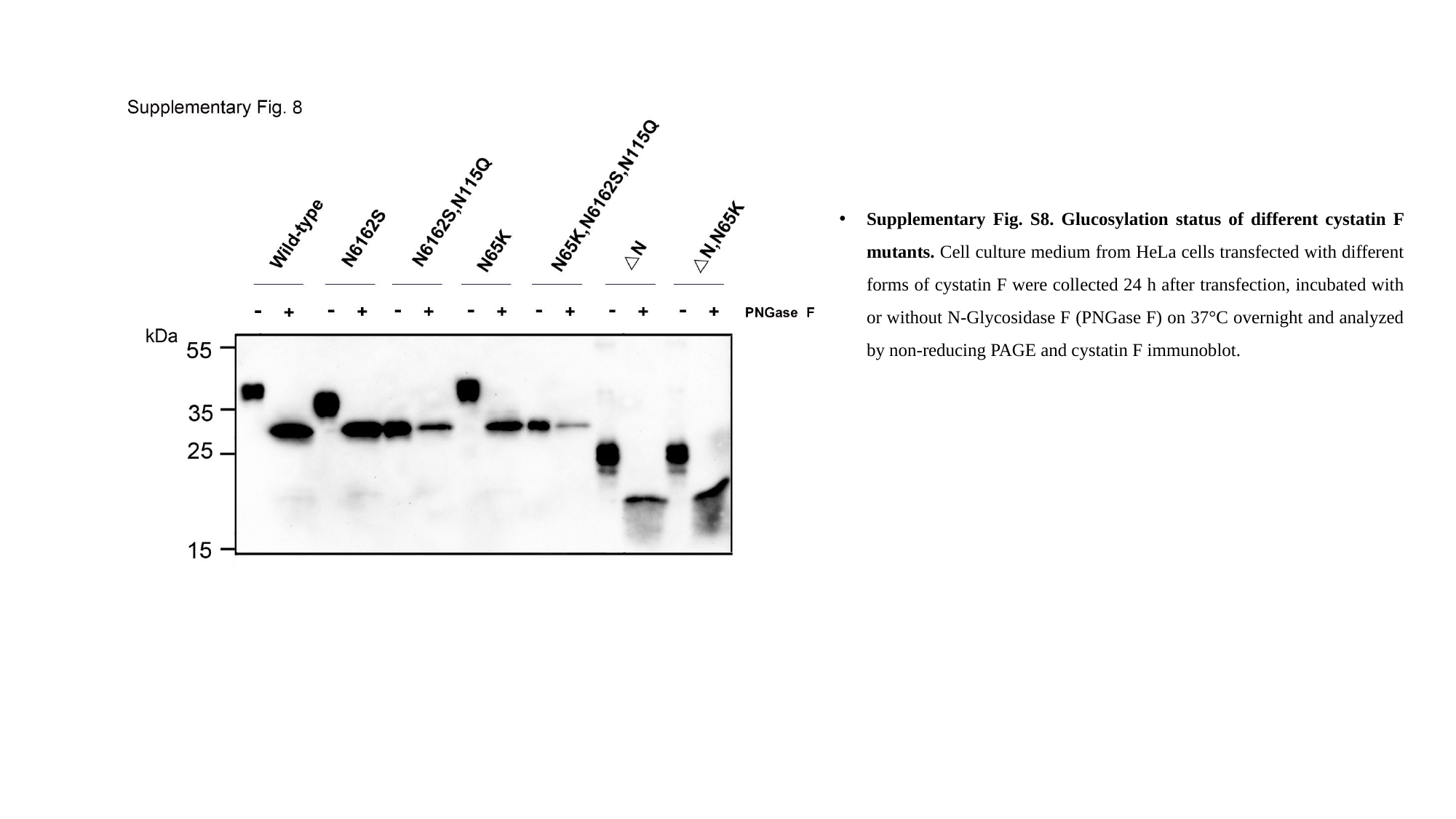

Supplementary Fig. S8. Glucosylation status of different cystatin F mutants. Cell culture medium from HeLa cells transfected with different forms of cystatin F were collected 24 h after transfection, incubated with or without N-Glycosidase F (PNGase F) on 37°C overnight and analyzed by non-reducing PAGE and cystatin F immunoblot.
